# Targeting microRNA-145-mediated progressive phenotypes of early bladder cancer in a molecularly defined *in vivo* model

**DOI:** 10.1016/j.omtn.2023.06.009

**Published:** 2023-07-03

**Authors:** Kazuki Heishima, Nobuhiko Sugito, Chikara Abe, Akihiro Hirata, Hiroki Sakai, Yukihiro Akao

**Affiliations:** 1 The United Graduate School of Drug Discovery and Medical Information Sciences, Gifu University, Gifu, Gifu, Japan; 2 Institute for Advanced Study (GUiAS), Gifu University, Gifu, Gifu, Japan; 3 Center for One Medicine Innovative Translational Research (COMIT), Gifu University, Gifu, Gifu, Japan; 4 Department of Physiology, Gifu University Graduate School of Medicine, Gifu, Gifu, Japan; 5 Laboratory of Veterinary Pathology, Joint Department of Veterinary Medicine, Faculty of Applied Biological Sciences, Gifu University, Gifu, Gifu, Japan

**Keywords:** MT: Non-coding RNAs, early bladder cancer, non-muscle-invasive bladder cancer, NMIBC, microRNA, microRNA-145, early bladder cancer rat model, intravesical therapy

## Abstract

A progressive subclass of early-stage non-muscle-invasive bladder cancer (NMIBC) frequently recurs and progress into invasive carcinoma, thus decreasing the overall survival rate of NMIBC. However, therapeutic development for progressive NMIBC has been challenging due to the lack of molecularly validated *in vivo* models and agents targeting its genetic vulnerability. We herein molecularly characterized an interventional model of progressive NMIBC and revealed the principal functions and therapeutic potential of microRNA-145 (miR-145) in early bladder tumorigenesis. N-butyl-N-(4-hydroxybutyl)nitrosamine-induced premalignant lesions (BiPLs) in rats exhibited downregulated expression of miR-145 as well as highly similar mutation/expression profiles to those of the human progressive NMIBC subclass with the worst prognosis. The expression patterns of miR-145 inversely correlated with those of BC-related oncogenes in BiPLs. We also demonstrated that miR-145 dominantly regulated interferon pathways and c-Myc expression, which play a crucial role in the pathogenesis of progressive NMIBC. Furthermore, we demonstrated that miR-145 replacement with a novel miR-145-based intravesical agent (miR-145S1) significantly inhibited the progression of BiPLs *in vivo*. These results provide insights into the essential role of miR-145 as the earliest-acting oncogenic driver of bladder tumorigenesis as well as a validated interventional model and novel miR-145-based nucleic acid therapeutic agent for progressive NMIBC.

## Graphical abstract

**Figure:**
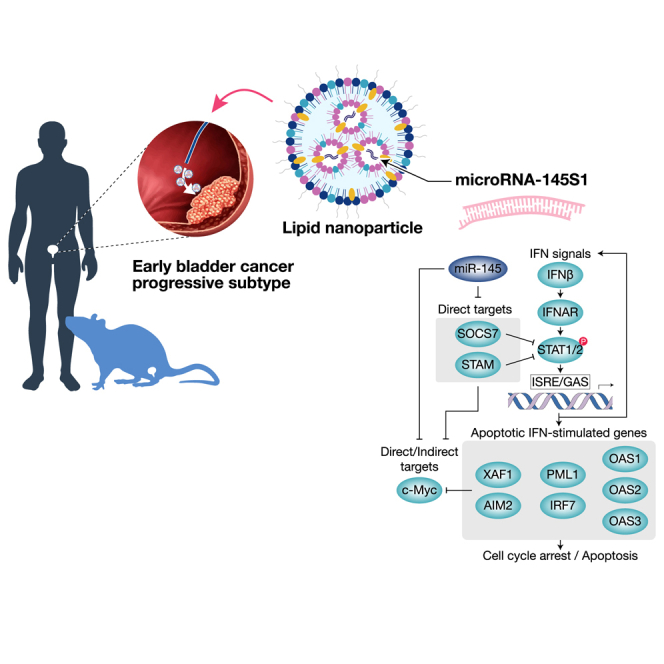

## Teaser

Heishima and colleagues revealed that N-butyl-N-(4-hydroxybutyl)nitrosamine-induced premalignant lesions in rats have mutation/expression profiles highly similar to those of early-stage non-muscle-invasive bladder cancer (NMIBC) with the worst prognosis. Among many genetic alterations in NMIBC, microRNA-145 is likely the principal regulator and the earliest-acting oncogenic driver of bladder tumorigenesis.

## Introduction

Bladder cancer (BC) is the most common urogenital cancer, with more than 430,000 new cases and 186,000 deaths being reported each year.^1^ Approximately 75% of patients are diagnosed and treated at an early stage of non-muscle-invasive bladder cancer (NMIBC).^2^ However, despite an early diagnosis and treatment, NMIBC recurs in 50%–70% of patients, with 10%–15% showing disease progression. The recurrence and progression of BC are mainly responsible for a progressive subclass of NMIBC, which is defined by specific molecular signatures (UROMOL2021; class 1, 2a, 2b, or 3)^3^ rather than the TNM subtype (Ta, papillary non-invasive carcinoma; T1, tumors infiltrating the lamina propria; Tis/CIS, carcinoma *in situ*). Class 2a progressive NMIBC has the worst prognosis due to its high rate of recurrence and progression into muscle-invasive bladder cancer (MIBC). Once these lesions progress to metastatic MIBC, the 5-year survival rate markedly decreases to 5%^4^ despite treatment with highly invasive radical cystectomy and urinary diversion. Therefore, the control of progressive NMIBC has attracted enormous interest in clinical fields.

The therapeutic option to treat progressive NMIBC is currently limited to the intravesical administration of Bacillus Calmette-Guérin (BCG), a non-specific immunogenic substance. Although intravesical immunotherapy with BCG has been the frontline therapeutic modality for decades, its non-specific targeting mechanism results in a high recurrence rate and toxicity. NMIBC recurs in more than 53.3% of patients receiving BCG within 3.6 years^5^ and more than 60% of patients experience various degrees of adverse effects, including chemical cystitis, irritative voiding symptoms, malaise, severe sepsis, and allergic reactions (skin rash, arthralgia, and reactive arthritis).^6^ Other treatment modalities, such as transurethral surgery and systemic chemotherapy, are less effective for progressive NMIBC, particularly CIS, because these lesions are challenging to identify and reside only in the mucosal layer, which is poorly vascularized. These properties indicate the vital necessity of developing a novel intravesical agent that can directly reach the urothelial mucosa and specifically target the genetic vulnerabilities of progressive NMIBC.

Major genetic alterations in BC include the dysregulation of numerous microRNAs (miRNAs), which are small non-coding RNAs that regulate the expression of multiple genes through RNA interference (RNAi).^7^ Among these alterations, the downregulation of microRNA-145-5p (miR-145) is one of the genetic hallmarks of BC, being evident from early-stage NMIBC to late-stage MIBC.^8^^,^^9^ miR-145 plays tumor suppressor roles in BC and functions as the central regulator to inhibit the expression of BC-associated oncogenes, including FSCN,^10^ c-Myc,^11^^,^^12^ PTBP1,^13^^,^^14^ IGF1R,^15^ and MDM2.^16^^,^^17^ These findings suggest a strong relationship between miR-145 expression and early bladder tumorigenesis.

Despite the implicated relationship, the pathogenic contribution and therapeutic applications of miR-145 for progressive NMIBC have not been examined in detail, mainly due to the lack of a validated/suitable model. The BC-miR-145 relationship has been investigated in cell lines^10^^,^^13^^,^^15^ and cell-derived xenograft models.^18^ However, these models exhibit aggressive/homogeneous growth in an immune-deficient microenvironment,^19^ in contrast to NMIBC, which exhibits slow/heterogeneous growth in an intact immune environment. Patient-derived xenograft models have the same defect as cell-derived xenograft models, and thus are also unsuitable as NMIBC models.^19^

Based on the characteristics of NMIBC, rat N-butyl-N-(4-hydroxybutyl)nitrosamine (BBN)-induced premalignant lesions (BiPLs) may serve as a suitable model of progressive NMIBC.^20^^,^^21^^,^^22^ BBN is a DNA alkylation compound derived from a chemical in cigarette smoke (N-nitroso-di-N-butylamine) and induces premalignant lesions that predominantly progress toward invasive carcinoma as a part of multiple carcinogenic steps.^23^^,^^24^ Rat BiPLs share the characteristics of human NMIBC in terms of the pathogenic factor (exposure to the cigarette smoke-derived chemical) and heterogeneous/slow growth in an intact immune environment.^23^^,^^24^ In addition, the rat BBN-induced BC model offers a larger body size and greater accessibility to the bladder than the mouse model, with resulting advantages in testing the efficacy of intravesical infusion therapy. However, the majority of studies using rat BBN-induced BC models have focused on late-stage lesions^21^^,^^25^ and not on early lesions, i.e., BiPLs. Therefore, limited information is currently available on the molecular characteristics and suitability of rat BiPLs as a model of human progressive NMIBC.

In this study, we molecularly characterized rat BiPLs in terms of somatic mutations, histopathology, and oncogene expression. We identified that rat BiPLs had high molecular similarity to the human progressive NMIBC subclass with the worst prognosis (UROMOL2021 class 2a). By using this molecularly defined model, we demonstrated that miR-145 predominantly regulated the major phenotypes of progressive NMIBC, which strongly suggests the involvement of miR-145 in the pathogenesis of BC. Furthermore, we developed a novel miR-145-based intravesical therapeutic agent and demonstrated its efficacy against progressive premalignant lesions *in vivo*.

## Results

### Expression of miR-145 in human and rat BC cells

The downregulation of miR-145 is a well-known genetic alteration in human BC. However, the association between miR-145 expression and the early pathogenesis of BC and its prognostic contribution remain unclear in human patients and BBN-induced rat models.

**Figure 1:**
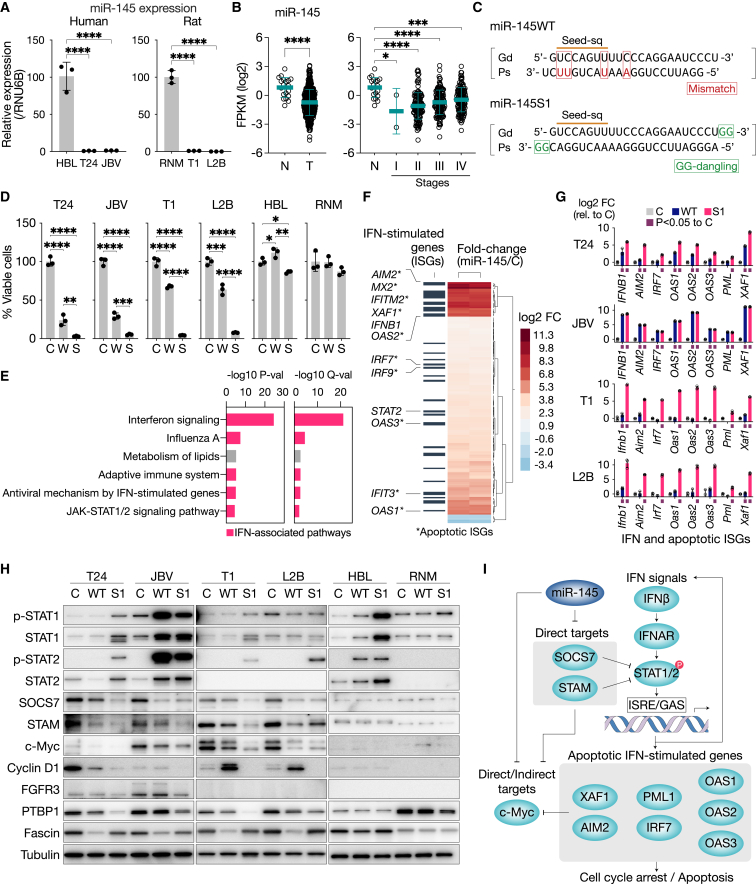
Expression and anti-tumor properties of miR-145 in human and rat bladder cancer cells (A) Expression levels of miR-145 in human and rat cell lines derived from BC or normal urothelial cells. Human BC cell lines, T24 and 253 JB-V (JBV); rat BBN-induced BC cell lines, NBT-T1 (T1) and NBT-L2B (L2B); normal diploid cells, HBlEpC (HBL, human) and RNM (rat, primary culture). (B) Expression levels of miR-145 in human clinical samples of different stages from TCGA. N, normal tissues; T, tumor tissues. (C) Double-stranded RNA sequences of miR-145 wild-type (miR-145WT) and miR-145S1 with full-match passenger sequences and GG dangling. (D) Viable cell percent among human and rat BC cells treated with miR-145WT (W), miR-145S1 (S), or control miRNA (C) at 20 nM for 72 h. (E) Pathways significantly altered in human BC cells (JBV) treated with 10 nM of miR-145WT or control miRNA for 48 h. A pathway enrichment analysis with Metascape: a p value (P-val) ≤ 0.01 and a q value (Q-val) ≤ 0.01. Differently expressed genes (DEGs): absolute FCs ≥ 1.5, p ≤ 0.05, and two-tailed, paired Student’s t test (n = 2). (F) A cluster analysis of DEGs with illustrations by heatmaps. Heatmaps are annotated with gray bars indicating interferon-stimulated genes (ISGs). (G) A qRT-PCR analysis of the expression of apoptotic ISGs in human and rat BC cells treated with 20 nM of miRNAs (W, S, or C) for 48 h. (H) An immunoblot analysis of IFN signaling pathways (p-STAT1/2, STAT1/2, SOCS7, and STAM) and miR-145-associated oncogenes (c-Myc, cyclin D1, FGFR3, PTBP1, and Fascin) in BC cells treated for 72 h with 20 nM of miRNAs (WT, S1, or C). (I) Scheme for the potential mechanism underlying miR-145-mediated anti-tumor effects. Data are presented as means ± SD. ∗p < 0.05, ∗∗p < 0.01, ∗∗∗p < 0.001, ∗∗∗∗p < 0.0001; one-way ANOVA with Dunnett’s post hoc test.

To address these issues, we initially investigated the expression levels of miR-145 in human and rat BC by utilizing BC cell lines and The Cancer Genome Atlas (TCGA)-registered BC clinical sample data. The expression of miR-145 was significantly downregulated in both human (T24 and 253JB-V) and rat (NBT-T1, NBT-L2B) BC cell lines compared with normal diploid urothelial cells (HBlEpC for humans and RNM for rats; Figures 1A and S1A). In addition, miR-145 was downregulated in human BC clinical samples registered in TCGA database (Figure 1B). The downregulation of miR-145 was observed at all clinical stages (Figure 1B), which is consistent with previous findings showing that its downregulation was already evident at the early stage of human BC.^8^

On the other hand, comparisons between cancer tissue samples showed that high miR-145 expression levels in samples were associated with advanced clinical stages and short survival times (Figures 1B and S1B). This result is in direct contrast to the well-known anti-tumor characteristics of miR-145.^10^^,^^13^^,^^15^^,^^18^ This inconsistency has been the subject of debate^8^^,^^26^^,^^27^; however, it may be attributed to contamination with smooth muscle, which strongly expresses miR-145.^28^ High-grade BC involves more stromal tissues containing smooth muscle or vascular smooth muscle (Figure S1C). The expression levels of miR-145 in clinical samples strongly correlated with those of smooth muscle (αSMA) and vascular markers (CD31; Figure S1D). The correlation between high miR-145 expression and an advanced stage and short survival time were eliminated when miR-145 levels were normalized to αSMA expression levels (Figures S1E and S1F). These results were also consistent with the observation that these contradictory associations (high miR-145 expression-poor prognosis) were only reported in studies that employed whole-tissue lysate-based methods to measure miR-145.^26^^,^^27^ In contrast, a study that used the *in situ* hybridization (ISH) technique, which distinguishes expression in urothelial cells from that in other cell types, reported that high miR-145 expression was associated with a favorable prognosis.^8^

Collectively, these results indicated that the downregulation of miR-145 was evident in both human and rat cell lines, as well as in human clinical samples at earlier stages. The previously reported association between high miR-145 expression and poor survival may have been due to contaminated tissues that abundantly express miR-145. Therefore, ISH appears to be a suitable method for evaluating miR-145 expression in urothelial tumor tissues.

### Anti-proliferative effects of miR-145 *in vitro*

To clarify the functions of miR-145 in bladder tumorigenesis, we examined the effects of wild-type miR-145 (miR-145WT, Figure 1C) on the proliferation and cell death of BC cells. The transfection of miR-145WT significantly decreased the viable cell count in both human (T24 and 253JB-V) and rat (NBT-T1 and NBT-L2B) cell lines (Figure 1D). The decrease observed in viable cells was due to cell-cycle arrest with the features of apoptosis, which is characterized by an increased sub-G fraction cell count and the slightly increased cleavage of PARP (Figures S2A–S2C). These results suggested that the replacement of miR-145 had anti-oncogenic properties in both human and rat BC cells.

In consideration of future clinical applications, we attempted to improve the efficacy of miR-145 by adding modifications to nucleotide sequences. We synthesized miR-145 derivatives (12 derivatives, named miR-145S1 to S12) with different chemical or sequence modifications (Figures S3A and S3B). Among these, miR-145S1, with a fully complementary passenger sequence (full-match sequence) and GG overhang at the 3′ terminus (GG dangling), exhibited the strongest growth inhibitory activity in a human BC cell line (T24; Figures 1C, S3A, and S3B). Similar to the transfection of miR-145WT, that of miR-145S1 decreased viable cell counts in both human and rat BC cells. However, the anti-tumor efficacy of miR-145S1 was significantly higher than that of miR-145WT (Figures 1D and S2A–S2C). The transfection of miR-145S1 induced a stronger degree of cell-cycle arrest and apoptotic cell death (Figures S2A–S2C). Affected cells showed the downregulation of a proliferation marker (p-Histone H3), an increased sub-G fraction cell count, and the cleavage of PARP and caspase-3 (Figures S2A–S2C). These results suggested that miR-145S1 has more favorable characteristics as a therapeutic agent. miR-145S1 and miR-145WT both exerted negligible effects on normal diploid cells (HBlEpC and RNM; Figures 1D and S2A–S2C), indicating the tumor specificity of miR-145 replacement therapy.

### Comprehensive analysis to identify functional target genes and pathways of miR-145

Although miR-145 has multiple target genes and pathways associated with bladder tumorigenesis, the mechanisms underlying its anti-tumor effects in BC have not yet been elucidated in detail. Therefore, we performed a transcriptome analysis to identify the main pathways contributing to the anti-tumor activity of miR-145 in BC.

We found that the dominant phenotype induced by the replacement of miR-145 was the upregulation of interferon (IFN)/JAK-STAT1/2 signaling and its associated pathways (Figures 1E and 1F). The “IFN signaling” pathway had the highest statistical significance (log10 p or q value less than −20), and most of the other enriched pathways were also associated with IFN signaling (5 out of 6 pathways; Figure 1E). The majority of differently expressed genes (DEGs) were upregulated (143 out of 148 genes, 96.6%; Figure 1F), whereas few were downregulated (5 out of 148 genes, 3.4%). Most of the upregulated DEGs were STAT1/2-regulated apoptotic IFN-stimulated genes (ISGs), such as *IFNB1* (IFNβ), *AIM2*, *XAF1*, and *OAS1* (Figure 1F). Genes regulated by other members of the STAT family, such as STAT3, were not significantly enriched in DEGs, suggesting that miR-145 exerted its anti-tumor functions mainly through STAT1/2 transcriptional factors rather than through other members of the STAT family. Furthermore, a qRT-PCR analysis showed that the replacement of miR-145 significantly upregulated apoptotic ISGs (*IFNB1*, *AIM2*, *IRF7*, *OAS1*, *OAS2*, *OAS3*, *PML*, and *XAF1*, as well as their rat homologs) in all of the human/rat cell lines evaluated (Figure 1G). The expression levels of ISGs were up to approximately 1,000-fold higher in cells treated with miR-145S1 than in steady-state cells (Figure 1G), and its efficacy was markedly stronger than that of miR-145WT.

Consistent with this result, an immunoblot analysis showed that human/rat BC cells transfected with miR-145S1 strongly induced the activation of IFN-STAT1/2 signaling (the upregulation of p-STAT1/2 and STAT1/2; Figure 1H), albeit with weak effects on some cells treated with miR-145WT. The replacement of miR-145 also induced the concomitant downregulation of other oncogenes related to BC, including cyclin D1, FGFR3∗, Fascin∗, c-Myc∗, and PTBP1∗ (∗direct targets of miR-145; Figure 1H). These results suggested that miR-145 exerted its anti-tumor effects by targeting multiple oncogenes, in addition to its strong efficacy in activating the IFN-STAT1/2 pathways.

To assess the functional roles of STAT1/2 in bladder tumorigenesis in humans, we investigated the association between overall survival and STAT1/2 expression in clinical samples from BC patients using TCGA database. The results showed that the downregulation of STAT1/2 significantly correlated with unfavorable outcomes in human BC (Figure S4A). The results also suggested that the miR-145-STAT1/2 axis was closely linked to the tumorigenesis and malignant progression of BC and that the interventional upregulation of the miR-145-STAT1/2 axis may exert anti-tumor effects in human BC patients.

We then attempted to elucidate the role of miR-145 in the upregulation of components of the IFN-STAT1/2 pathways. A previous study reported that miR-145 induced the activation of IFN via the RNAi-mediated inhibition of *SOCS7*, a negative regulator of IFN-STAT1/2 signaling, and these effects did not occur via the non-specific IFN activation induced by double-stranded RNA.^29^ Consistent with these findings, an immunoblot analysis showed that the replacement of miR-145 downregulated the expression of SOCS7 in human/rat BC cells (Figure 1H). Furthermore, the computational analysis identified another IFN suppressor, *STAM* as an unreported potential direct target of miR-145. Since *STAM* has not been validated as a direct target of miR-145, we performed a luciferase reporter assay to establish whether miR-145 directly bound to the 3′ UTR of *STAM* (Figure S4B). The transfection of miR-145WT or miR-145S1 significantly suppressed luciferase activity, and this suppression of luciferase activity was successfully canceled in cells expressing the mutated binding site (Figure S4C). Therefore, *STAM* was verified as a direct target gene of miR-145 (Figures S4B and S4C), suggesting that miR-145 directly inhibited multiple IFN-STAT1/2 suppressors to induce the strong activation of the IFN-STAT1/2 pathways. To examine the roles of SOCS7 and STAM in the IFN-STAT1/2 pathway and c-Myc expression, we performed siRNA-based knockdown in BC cells highly expressing c-Myc (human, JBV; rat, L2B). The results showed that the siRNA-mediated knockdown of SOCS7 or STAM induced a significant decrease in viable cells and the upregulation of STAT1/2 (Figures S4D and S4E). Notably, the knockdown of SOCS7 or STAM markedly downregulated the expression of c-Myc (Figure S4E), suggesting that the effects of miR-145 on c-Myc expression were through a synergetic mechanism involving both RNAi-mediated direct targeting^11^^,^^12^ and an IFN-STAT1/2-mediated indirect pathway. These results were consistent with previous findings showing that IFN signaling negatively regulated c-Myc expression.^30^^,^^31^ The downregulation of c-Myc may not be a result of the downregulation of the PI3K/Akt pathway because p-Akt expression did not correlate with the downregulation of c-Myc in BC cells following the knockdown of SOCS7 or STAM (Figure S4E).

In most cases, the efficacy of miR-145S1 to regulate these oncogenes was higher than that of miR-145WT, and miR-145S1 and miR-145WT both induced similar molecular changes in human and rat BC cells. These results suggested that miR-145S1 possessed strong anti-tumor properties and that the modifications added to miR-145S1 did not markedly affect the spectrum of the target genes, albeit with some exceptions. These exceptions included cyclin D1, which was downregulated by the transfection of miR-145 under most conditions, but was upregulated only in rat BC cells treated with miR-145WT. In addition, FGFR3 was downregulated in human BC cells transfected with miR-145WT or miR-145S1; however, FGFR3 was not expressed in rat BC cells. This result was consistent with previous findings showing that the rat BBN-induced BC model typically lacked the expression of FGFR3.^32^

The normal urothelial cells had only weak expressions of c-Myc, cyclin D1, and FGFR3, and both miR-145WT and miR-145S1 exerted minimal effects on these normal urothelial cells. As an exception, STAT1/2 signaling was upregulated in a human normal urothelial cell line (HBL).

Collectively, these results suggest that the replacement of miR-145 exerts anti-proliferative effects on BC cells through the regulation of the IFN-STAT1/2 pathway and also that the underlying mechanisms appear to be shared between humans and rats (Figure 1I). Furthermore, miR-145S1 displayed a similar target gene spectrum, but exerted more potent inhibitory effects on its targets than miR-145WT.

### Gross and histopathological characterization of rat BiPLs

The above-described *in vitro* results suggested that miR-145 plays essential roles in the pathogenesis of BC and may be targeted as a therapeutic strategy for BC. Therefore, we conducted *in vivo* studies using a rat BBN-induced BC model to assess the potential roles of miR-145 in the regulation of the progressive premalignant phenotypes.

BBN-initiated rats develop premalignant lesions, i.e., BiPLs, which sequentially progress toward invasive malignant tumors. Rat BiPLs show a papillary growth pattern that progresses from papillary/nodular (PN) hyperplasia to invasive carcinoma, which differs from that of human CIS (the major phenotype of progressive NMIBC) showing a flat growth pattern that evolves toward invasive carcinoma.^23^^,^^24^ However, the majority of rat BiPLs progress toward invasive carcinoma,^23^^,^^24^ and their strong progressive predisposition is rather similar to human CIS than human Ta tumors, which have a similar morphology but less potential to progress toward invasive carcinoma. Despite morphological differences, the BiPL model may serve as a model of human progressive NMIBC, given its strong progressive predisposition.^23^^,^^24^ However, previous studies using BBN-induced rat models mainly focused on advanced/late-stage cancers; therefore, early bladder lesions, i.e., BiPLs, have not yet been characterized in detail, and the molecular similarities between rat BiPLs and human progressive NMIBC, as well as their utility as a model, remained unknown.

To establish whether the BiPL model serves as a model of human progressive NMIBC, we assessed its histopathological and molecular characteristics. A recent study classified human NMIBCs into four subclasses with different prognoses and recurrence rates based on their mutation/expression profiles (UROMOL2021; class 1, 2a, 2b, or 3).^3^ The study identified that the molecular profiles were more predictive for the prognosis and recurrence rates of NMIBC, rather than the conventional morphological characteristics or stage (Ta, T1, or CIS).^3^ Therefore, we focused on investigating class-defining molecular markers or signatures that may be conserved between humans and rats.

**Figure 2:**
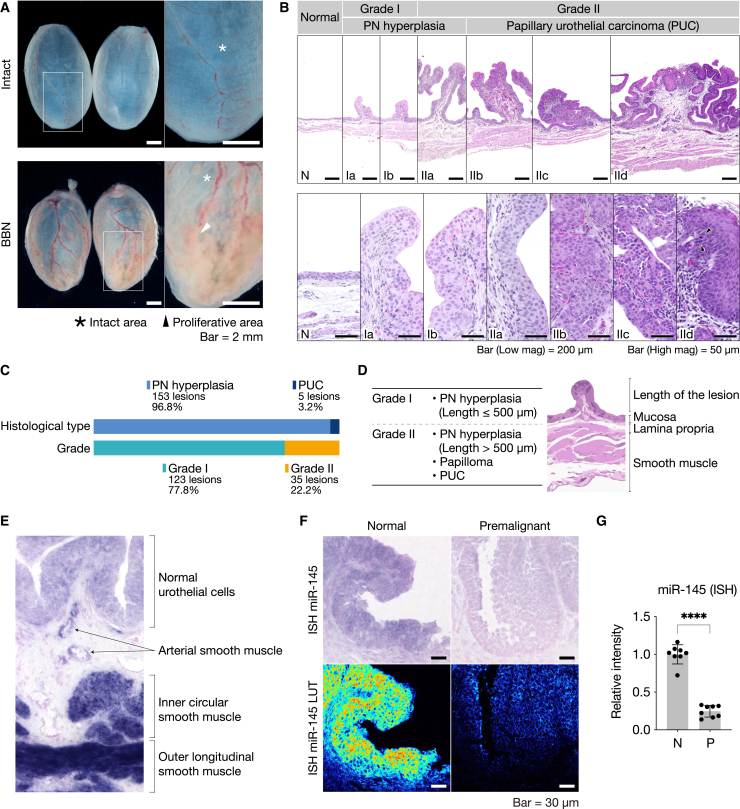
Pathological characterization, grading, and miR-145 expression of rat BiPLs (A) Representative gross images of bladders treated with BBN (BBN) or not exposed to BBN (intact). Stars and arrowheads indicate intact and hyperplastic areas, respectively. Scale bars, 2 mm. (B) H&E images and the histopathological grading of lesions in the rat BiPL model. Lesions included papillary/nodular hyperplasia (PN hyperplasia) and papillary urothelial carcinoma (PUC). Lesions were classified into three grades as follows: N, no morphologically abnormal proliferation; grade I, early PN hyperplasia with a lesion length of less than 500 μm; grade II, late PN hyperplasia with a lesion length of greater than 500 μm and PUC. Scale bars, 200 μm for low-magnification (low mag) or 50 μm for high-magnification (high mag) images. (C) The counts and percentages of each histological type and grade observed in this study. (D) Definition of the grading system for rat BiPL in this study. (E) Representative images of *in situ* hybridization (ISH) for miR-145 in normal bladder tissue. Signals were visualized in blue using the ALP-NBT/BCIP system with red counterstaining with Nuclear Fast Red. (F) Representative images of miR-145 ISH in BiPLs and normal bladder tissues. A color lookup table (LUT) was used to illustrate NBT-BCIP signal intensities; high and low expression levels are colored in red and blue to black, respectively. Scale bars, 30 μm. A detailed version is available in Figure S5B. (G) Relative ISH staining intensities of miR-145. N and PL indicate normal and BiPL tissues, respectively.

BiPLs were generated by the free intake of water containing 0.05% BBN and 0.005% Tween 80 for 16 weeks. After an additional 2 weeks without BBN exposure, rats at 18 weeks exhibited mild urothelial proliferation in the bladder (Figure 2A). To perform consistent gross and histopathological evaluations, bladder samples were fixed *in situ* by a uniform infusion of 300 μL of 4% paraformaldehyde into the bladder and were then sliced in the sagittal plane. Urothelial proliferation was grossly observable in *in-situ*-fixed samples, but was difficult to identify in samples processed under the normal fixation protocol (Figures 2A and S5A).

Histopathologically, model rats developed PN hyperplasia and papillary urothelial carcinoma (PUC) (Figure 2B). PN hyperplasia was predominantly observed, accounting for 96.8% of the total lesion count (Figure 2C). A few PUC lesions (3.2% of the total) were observed; however, they were early-stage non-invasive PUC with limited malignant areas. CIS was not identified in the rat model, which is consistent with previous findings showing that the BBN-induced rat model rarely develops CIS.^21^ These results indicated that rat BiPLs have similar morphological characteristics to those of human Ta tumors, as described above.

We classified lesions into three grades based on their histopathological features and used them for morphological evaluation in this study (Figure 2D). The grading system used was as follows: N, normal; grade I, early PN hyperplasia with a lesion length of less than 500 μm; grade II, late PN hyperplasia with a lesion length of greater than 500 μm or PUC. According to the grading system, we identified 123 grade I and 35 grade II lesions (grade I/II ratio, 0.28) as the total lesion count in 9 rats (Figure 2C).

### Downregulation of miR-145 in rat BiPLs

The downregulation of miR-145 is a hallmark of bladder tumorigenesis evident from the early stage in humans^9^; however, the expression status of miR-145 and its association with bladder tumorigenesis in rats remained unclear. Therefore, we examined the expression status of miR-145 in rat BiPLs.

Rats initiated by BBN developed multiple premalignant lesions of different stage. The lesions were very near to each other and adjacent to normal urothelial cells. A PCR-based assessment using bulk tissue lysates was not suitable for a reliable analysis because the samples could have been contaminated with lesions of different grade, normal urothelial cells, or smooth muscle tissues highly expressing miR-145. To analyze the expression of miR-145 with good spatial resolution, we utilized the miRNA ISH technique to detect miR-145 molecules in heterogeneous samples.

miR-145 ISH showed that normal urothelial cells had the expression of miR-145, which was observed as a finely stippled blue-positive stain in the cytoplasm or occasionally in nucleoli under NBT/BCIP staining (Figure 2E). The expression of miR-145 was already downregulated in PN hyperplasia cells (“Premalignant” in Figures 2F and 2G) compared with adjacent urothelial cells with normal morphological characteristics (a uniform number of urothelial cell layers with no nuclear/cytoplasmic atypia) and not expressing premalignant markers (Figure S5B). The results suggested that the downregulation occurred in the early stage of tumorigenesis, similar to its timing in human BC. Moreover, miR-145 was highly expressed in arterial smooth muscle and inner circular and outer longitudinal smooth muscle (Figure 2E), which is consistent with previous findings.^28^ Expression levels were markedly higher in smooth muscle than in normal urothelial cells; therefore, contamination with smooth muscle tissues may obscure actual differences between BiPL cells and normal urothelial cells. We also performed an miRNA qRT-PCR analysis using bulk tissue lysates, but found no significant differences between BiPL and normal urothelial samples as predicted (Figure S5C).

### Genetic anomalies in rat BiPLs

To further characterize rat BiPLs, we assessed single base substitution (SBS) mutation signatures, somatic mutations, copy number variants (CNVs), and structural variants (SVs) using next-generation sequencing (NGS). Three rat BiPL tissues (P1-3) and rat normal urothelial tissues (N) from a BBN-treated bladder were sampled under a stereomicroscope. Rat BiPL-associated genetic anomalies were identified by comparing rat BiPL and normal urothelial tissues. The mutation profiles of rat BiPLs were compared with those of human NMIBCs (UROMOL2016/2021).^3^^,^^33^

**Figure 3:**
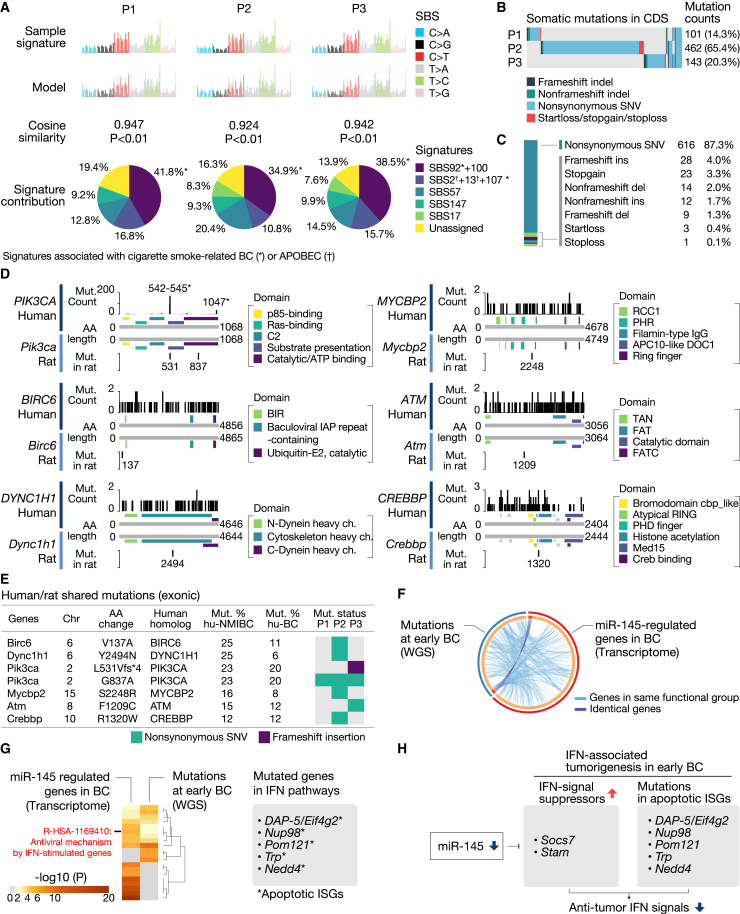
Mutation analyses of rat BiPLs (A) Comparisons of single base substitution (SBS) mutation signatures between rat BiPLs and human BC. “Sample” shows the profiles of BiPL SBS signatures. “Model” shows the profiles built by proportionally combining reported human BC signatures. Sample-model cosine similarities (highest at 1, lowest at −1), signature contributions, and the statistical significance were calculated with Signal. The signatures associated with cigarette smoke exposure and APOBEC are marked with asterisks (∗) and daggers (†), respectively. (B) Types, counts, and distributions of exonic mutations in each BiPL sample. (C) Counts and percentages of each type of exonic mutation. (D) Comparative mutation maps indicating the locations of the mutations identified in rat BiPL and human BC samples with information about the adjacent domains. Pathogenic mutations from human BC clinical samples were obtained from COSMIC. Asterisks (∗), hotspots of *PIK3CA*. (E) A list of exonic mutations shared between human BC and rat BiPLs. The mutation percentages in human NMIBC (Mut. % hu-NMIBC) and BC (Mut. % hu-BC) were obtained from the study by Hedegaard et al. and TCGA, respectively. SNV, single nucleotide variant. (F) Circos plot showing the functional overlap of mutations at early BC and miR-145-regulated genes. The strings connecting the circle-shaped bars show the association between genes mutated in early BC (from whole-genome sequencing [WGS]) and genes regulated by miR-145 (from a transcriptome analysis). The purple and light blue strings connect identical genes and genes in the same functional group, respectively. (G) Heatmaps showing overlaps in the significantly enriched pathways “mutations at early BC” and “miR-145-regulated genes.” A detailed version is available in Figure S6B. (H) Scheme showing the potential mechanism underlying IFN-associated tumorigenesis in early BC.

The SBS mutation signatures of rat BiPL samples showed significant similarities to those of human BC, with a cosine similarity of greater than 0.92 (p < 0.01; cosine similarity: highest at 1, lowest at −1; Figure 3A). In addition, the SBS signatures of BiPLs showed significant similarities to the mutation signatures associated with cigarette smoke-related BC (Figure 3A), indicating that the mutational signatures of BiPL mimicked those of the most prevalent phenotype in human NMIBC. Notably, rat BiPLs had APOBEC mutation signatures (Figure 3A), which are class-defining markers for the progressive NMIBC subclass with the worst prognosis (UROMOL2021 class 2a),^3^ demonstrating the similar mutational characteristics between rat BiPLs and human progressive NMIBC.

Rat BiPLs had different types and counts of somatic mutations in their coding sequences (exonic mutations; Figures 3B and 3C). The most frequent mutation type was non-synonymous single-nucleotide variants (87.3%), and mutations leading to substantial changes in the protein structure (frameshift indel, startloss, stopgain, and stoploss mutations) accounted for 9.1% of the total (Figure 3C). These mutations frequently occurred on chromosomes 7, 12, 15, and 20 (mutation frequency per chromosome, densely mutated region counts per chromosome; Figure S6A), consistent with the chromosomal aberrations observed in human BC.^34^^,^^35^^,^^36^^,^^37^ In particular, the result showing frequent mutations on chromosome 15 in rat BiPLs was consistent with the observation that genetic variations on chromosome 15 correlated with the risk of cigarette smoke-associated BC in humans.^36^

BiPLs harbored mutations in the homologs of frequently mutated genes in human NMIBC (*Birc6*, *Dync1h1*, *Pik3ca*, *Mycbp2*, *Atm*, and *Crebbp*; Figures 3D and 3E). Notably, we identified mutations in *Pik3ca*, one of the most frequently mutated oncogenes in human NMIBC, with mutation sites located near the hotspots of activating *PIK3CA* mutations in humans (Figures 3D and 3E).^38^ However, no mutations were observed in *Fgfr3*, which is consistent with previous findings showing that rat BBN-induced tumors did not harbor *Fgfr3* mutations.^32^

Of note, a pathway enrichment analysis using Metascape revealed that the functional spectrum of mutated genes highly overlapped with that of miR-145-regulated genes (Figure 3F). We found that the overlapped genes were enriched in the IFN-STAT1/2-associated pathway (Figure 3G). We also identified the mutations in crucial apoptotic ISGs, including *DAP-5*/*Eif4g2*, *Nup98*, *Pom121*, *Trp*, and *Nedd4* (Figure 3G). These results suggested that mutations in the early stage and the downregulation of miR-145 cooperatively contributed to the core mechanism of IFN-STAT1/2-mediated bladder tumorigenesis and also that miR-145 is the key targetable gene to treat early BC (Figure 3H).

Several CNVs were identified in BiPLs; however, fold changes were small (less than 2; Figures S6B–S6D). These CNVs were mainly associated with the metabolism of arachidonic acid (Figures S6E and S6F), suggesting that they were also associated with changes in the regulation of inflammation in BiPLs. No significant SV was detected in BiPL samples.

### Gene expression profiles of rat BiPLs

**Figure 4:**
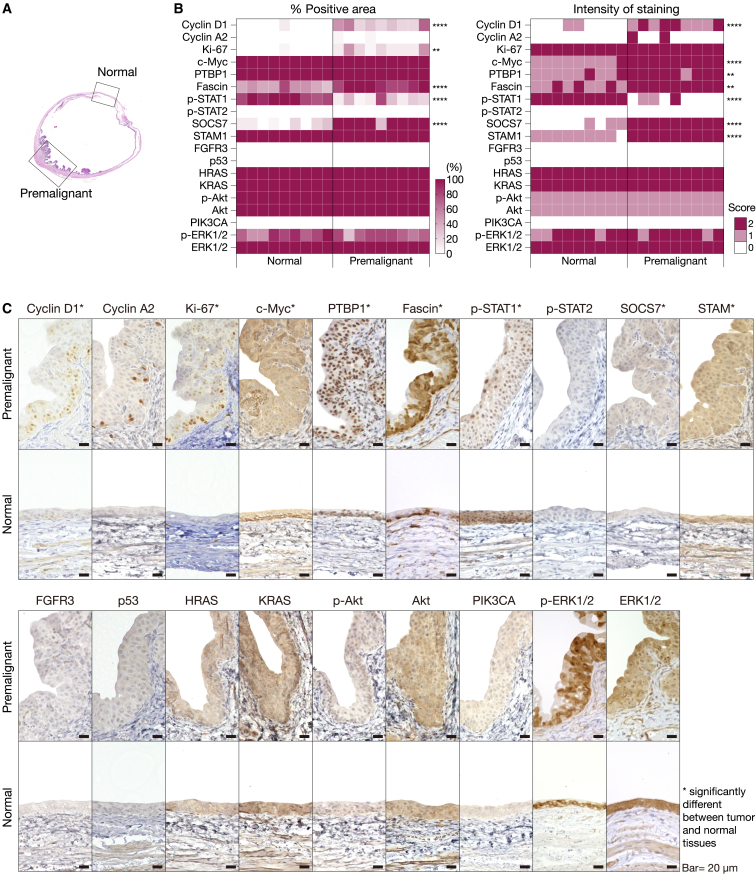
Expression of bladder cancer-associated markers in rat BiPLs (A) Representative H&E images of the areas of BiPLs (labeled “Premalignant”) at 18 weeks and of morphologically normal urothelial tissues (labeled “Normal”). (B and C) Heatmaps (B) and representative immunohistochemical images (C) showing the different expression of BC-associated markers in BiPL (Premalignant) and normal urothelial tissues (Normal). Scale bars, 20 μm. ∗∗p < 0.01, ∗∗∗p < 0.001, ∗∗∗∗p < 0.0001; two-tailed unpaired Student’s t test (n = 8).

**Figure 5:**
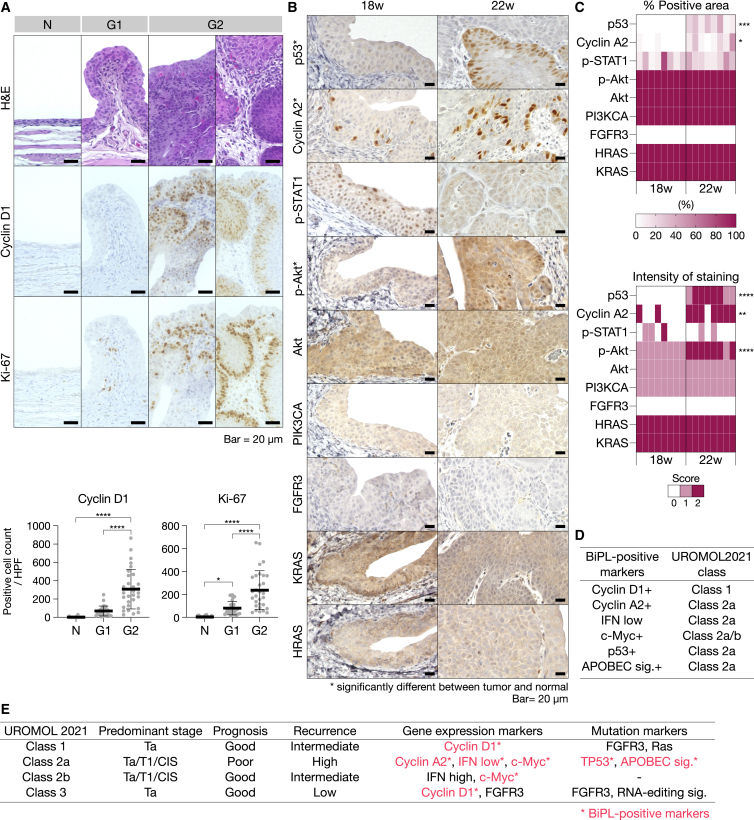
Progression-dependent changes in bladder cancer-associated marker expression in BiPLs (A) Histopathological grading-dependent changes in the expression of cyclin D1 and Ki-67 in BiPLs and normal tissues. Representative H&E and IHC images of BiPL samples of different histopathological grades and normal tissues are shown, along with the counts of cells positive for cyclin D1 and Ki-67. N, normal; G1, grade I; G2, grade II. Scale bars, 20 μm. Data are presented as means ± SD. ∗∗∗p < 0.001, ∗∗∗∗p < 0.0001; one-way ANOVA with Dunnett’s post hoc test. (B and C) Representative IHC images (B) and heatmaps (C) showing time course changes in disease marker expression in BiPL samples. N, normal; 18w, BBN-induced tumor development for 18 weeks; 22w, BBN-induced tumor development for 22 weeks. ∗p < 0.05, ∗∗p < 0.01, ∗∗∗p < 0.001, ∗∗∗∗p < 0.0001; two-tailed unpaired Student’s t test. (D) A list of markers with positive expression in BiPL (BiPL-positive markers) and their corresponding UROMOL2021 subclasses. (E) Summary of the UROMOL 2021 classification system and the markers used to distinguish the classes in this study. Asterisks (∗) indicate markers with positive expression in BiPL.

To assess the expression profiles of rat BiPLs, we immunohistochemically stained BiPL tissues (18 or 22 weeks after the start of BBN exposure) for BC-associated oncogenic proteins. These proteins were selected from the class-defining markers of the human NMIBC classification system (UROMOL2021)^3^ and were limited to the proteins likely conserved between humans and rats. Expression patterns were compared between BiPL cells (labeled “Premalignant”) and morphologically intact urothelial cells (labeled “Normal”; Figure 4A). The proteins examined included cell cycle markers (early cell cycle marker, cyclin D1; late cell cycle marker, cyclin A2; Ki-67), a p53 pathway component (p53), IFN-STAT1/2 pathway components (activated IFN markers, p-STAT1/2; IFN suppressors, SOCS7 and STAM), proteins involved in cell metabolism (c-Myc and PTBP1), a protein involved in cytoskeletal regulation (Fascin), and RTK-PI3K-RAS pathway components (FGFR3, PIK3CA, HRAS, KRAS, p-Akt, Akt, p-ERK1/2, and ERK1/2) in nine samples of each type (Figures 4B, 4C, and 5A–5C).

BiPLs in rats at 18 weeks had similar expression profiles to those of human class 2a NMIBC (c-Myc+, cyclin A2+/−, p-STAT1−, FGFR3−) with a slight mixture of class 1 and class 3 phenotypes (cyclin D1+, p53−; Figures 4B and 4C). Most BiPLs exhibited a further enhanced class 2a phenotype at 22 weeks (increased positivity for cyclin A2, p53, and p-Akt with the low expression of p-STAT1; Figures 5B and 5C). At 22 weeks, BiPLs were still negative for FGFR3, HRAS, KRAS, and PIK3CA (Figures 5B and 5C). These results indicated that most BiPLs had the class 2a-like phenotype (Figures 5D and 5E), i.e., the gene expression profile of the human progressive NMIBC subclass with the worst prognosis. In addition, the phenotype was consistent with previous findings showing that genetic anomalies in *Fgfr3*, *Hras*, and *Kras* were rare in rat BBN-induced BC models.^32^^,^^39^

Notably, these results demonstrated that the expression of cyclin D1 in rat BiPLs may serve as the earliest disease progression marker even though cyclin D1 is regarded as a marker for benign-oriented subclasses in the human NMIBC classification system (classes 1 and 3). The earliest lesions in the BBN-induced model were positive for cyclin D1 (Figure 5A), and cyclin D1-positive cell counts significantly increased as the grade became higher (Figure 5A). This result is consistent with previous findings showing that cyclin D1 is the definitive disease progression marker that distinguishes the premalignant lesions that eventually progress to invasive carcinoma in the rat BBN-induced model.^39^ Furthermore, comparisons between the expression of cyclin D1 and Ki-67 in serial sections revealed that many premalignant urothelial cells showed a cyclin D1+/Ki-67− profile, whereas the number of cyclin D1+/Ki-67+ cells was small and limited to the area close to the basement membrane (Figure 5A). These results suggested that cyclin D1 was overexpressed prior to cell cycle entry (Ki-67 overexpression); thus, the upregulation of cyclin D1 was not just a consequence of cell proliferation but an essential event triggering early bladder tumorigenesis in the rat BiPL model.

We also found that many direct targets of miR-145 (Fascin, SOCS7, STAM, c-Myc, and PTBP1) were upregulated in BiPLs, as demonstrated by the positive-stained area (Fascin) or staining intensity (SOCS7, STAM, c-Myc, and PTBP1; Figures 4B and 4C). The expression patterns of Fascin, SOCS7, and STAM were consistent with those in human BC samples (Figure S7). Notably, BiPLs exhibited the co-overexpression of c-Myc and cyclin D1, implying the contribution of the miR-145-c-Myc-cyclin D1 axis to the pathogenesis of premalignant lesions. Moreover, BiPLs showed the suppression of IFN-STAT1/2 signaling (downregulation of p-STAT1; Figures 4B and 4C), which was highly consistent with the downregulation of miR-145 and overexpression of IFN suppressor genes (SOCS7 and STAM) in BiPLs. In addition, since the low expression of IFN and high expression of c-Myc are class-defining markers for UROMOL2021 class 2a, this result further supported the hypothesis that the main phenotypes of rat BiPL are highly similar to those of human progressive NMIBC subclass with the worst prognosis (Figures 5D and 5E).

Collectively, these results indicated that rat BiPLs had similar profiles to those of UROMOL2021 class 2a human NMIBC, suggesting the suitability of the BiPL model as an interventional model for predicting therapeutic effects on human progressive NMIBC. In addition, the uniform upregulation of miR-145 target genes was consistent with the downregulation of miR-145, suggesting the essential involvement of miR-145 in bladder tumorigenesis.

### Correlations between miR-145 and disease marker expression

**Figure 6:**
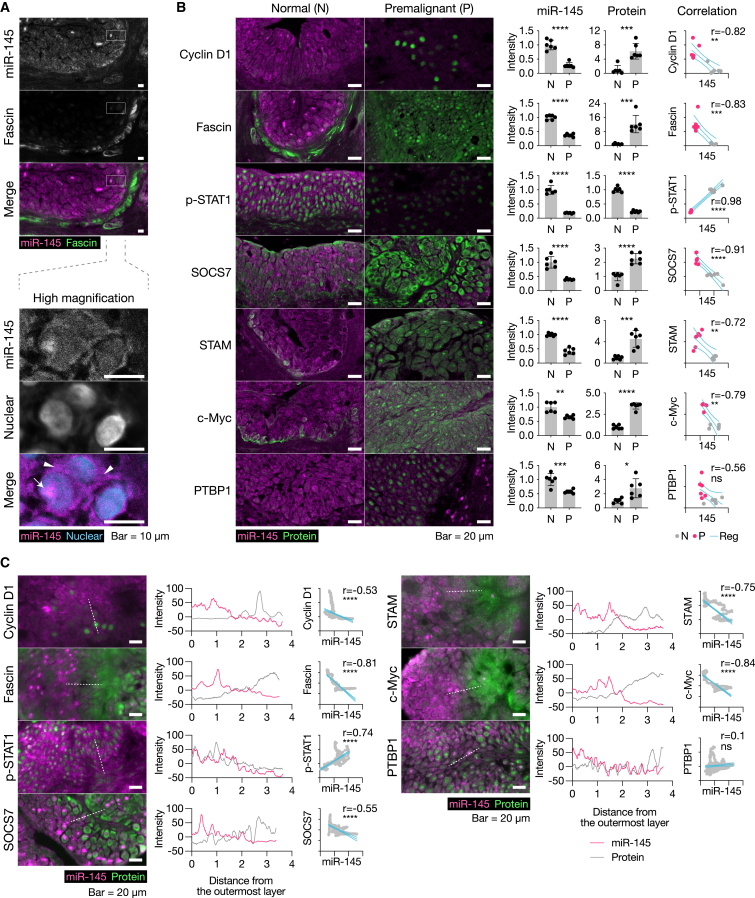
Co-localization of miR-145 and bladder cancer-associated protein expression in rat BiPLs (A) Representative images of fluorescence double ISH staining for miR-145 and IHC staining for Fascin (a miR-145 direct target gene) in morphologically normal urothelial tissue samples. High-magnification images show the localization of miR-145 expression in urothelial cells. The arrow and arrowheads indicate signals in the nucleolus and cytoplasm, respectively. Scale bars, 10 μm. (B) Representative images of fluorescence double ISH staining for miR-145 and IHC staining for BC-associated markers. Signal intensities and correlations are shown along with images of ISH-IHC double staining. N, normal tissue; P, premalignant tissue. Scale bars, 20 μm. Data are presented as the means ± SD (n = 8). ∗p < 0.05, ∗∗p < 0.01, ∗∗∗p < 0.001, ∗∗∗∗p < 0.0001; two-tailed unpaired Student’s t test to compare the signal intensities, Pearson’s correlation coefficient to test correlations. (C) Representative images showing the intralesional gradation of miR-145 and BC-associated marker expression. Line profiles and scatterplots to test correlations between miR-145 expression and protein expression are shown with images of ISH-IHC double staining. Scale bars, 20 μm. ∗p < 0.05, ∗∗p < 0.01, ∗∗∗p < 0.001, ∗∗∗∗p < 0.0001; Pearson’s correlation coefficient to test correlations.

We examined the co-localization of miR-145 with disease markers in BiPLs using miRNA ISH-immunohistochemical (IHC) double staining. miRNA ISH-IHC double staining followed by confocal microscopy successfully demonstrated that the expression of miR-145 was inversely correlated with that of the well-known direct target protein, Fascin, in normal urothelial tissues (Figure 6A). High-magnification images showed that miR-145 molecules were mainly localized to the cytoplasm and occasionally to nucleoli, but were less prevalent in the nucleoplasm (Figure 6A).

In addition, BiPLs with the low expression of miR-145 exhibited the significant upregulation of BC-related oncogenes (cyclin D1, Fascin∗, SOCS7∗, STAM∗, c-Myc∗, and PTBP1∗; ∗ indicates a direct target of miR-145) and a decreased level of a marker of IFN pathway activation (p-STAT1, Figures 6B and S8). Notably, normal urothelial cells showed the opposite pattern to that of BiPLs. A correlation analysis revealed that the expression of miR-145 inversely correlated with that of all BC-related oncogenes, except for PTBP1, between BiPL and normal urothelial cells (Figures 6B and S8).

Areas with different degrees of the downregulation of miR-145 were identified within BiPLs; thus, we investigated whether the intralesional gradation of miR-145 expression levels also correlated with the expression levels of disease markers. Due to the low baseline expression of miR-145 in BiPL, we utilized conventional fluorescence microscopy, which has a higher dynamic range than confocal microscopy, to visualize the subtle gradation of miR-145 expression. miR-145 expression patterns inversely correlated with those of all disease markers, except for PTBP1, within a single lesion (Figures 6C and S9).

These results suggested the prominent contribution of miR-145 to the pathogenesis of progressive premalignant lesions and the dysregulation of many BC-related genes *in vivo*, rationalizing the miR-145 replacement strategy for progressive NMIBC. In addition, the results indicated that the rat BiPL model mimicked the characteristics of the heterogeneity of human progressive NMIBC (UROMOL2021 class 2a), further demonstrating the advantage of using the rat BiPL model.

### *In vivo* delivery of miRNA to bladder urothelial cells

To assess the efficacy of miR-145 replacement therapy against progressive premalignant lesions *in vivo*, we performed the intravesical administration of miR-145 in the rat BBN-induced model.

**Figure 7:**
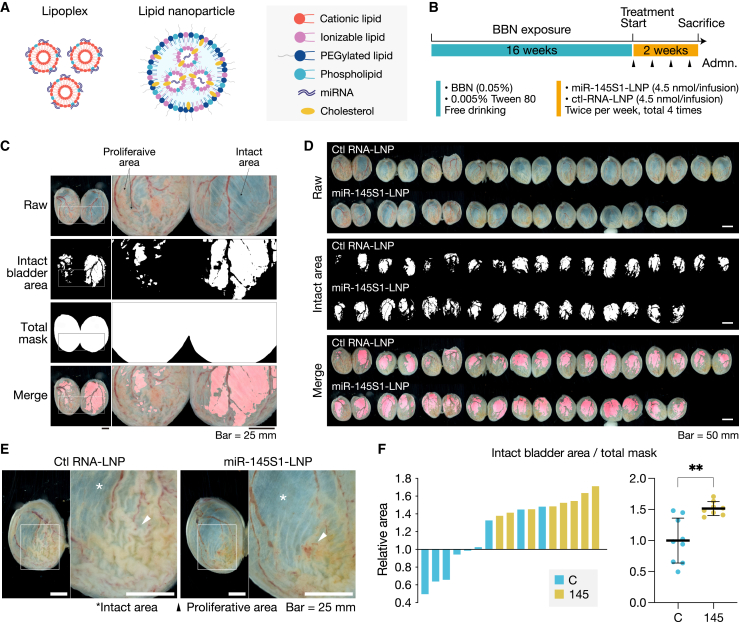
Gross analysis of the efficacy of miR-145 replacement for rat BiPLs (A) Illustration of the structure of Lipofectamine-based lipoplexes and lipid nanoparticles (LNPs). (B) Schematic illustration of the methods to establish the BBN-induced rat premalignant lesion model and treatment with miR-145S1-LNPs or ctl-RNA-LNPs. The triangles under the orange bar indicate the timing of administration (Admn). (C) Gross image analysis of the bladder. The intact bladder area was distinguished from the hyperplastic area based on color differences; e.g., the intact area had a thin bladder wall that was blue-black in color, whereas the hyperplastic area had a thick urothelial layer that was white to pink. The intact area was normalized to the total area of the bladder (Total mask). The actual intact area was matched with the calculated intact area in merged images. Scale bars, 25 mm. (D) All gross images and calculated intact areas of bladders treated with miR-145S1-LNPs (n = 8) or Ctl-RNA-LNPs (n = 9). Scale bars, 50 mm. (E) Representative high-magnification gross images of bladders treated with miR-145S1-LNP or ctl-RNA-LNP. The intact area and hyperplastic area are indicated with asterisks and arrowheads, respectively. Note that the bladder treated with miR-145S1-LNPs had a larger intact area (asterisks) than that treated with ctl-RNA-LNP. Scale bars, 25 mm. (F) Waterfall and swarm plot showing the relative intact bladder area per total mask in rats treated with miR-145S1-LNPs (n = 8) or Ctl-RNA-LNPs (n = 9). Data are presented as means ± SD. ∗∗p < 0.01; two-tailed unpaired Student’s t test.

We selected miR-145S1 as the therapeutic agent because of its higher efficacy than miR-145WT and thus more favorable properties for drug development. For the drug delivery system, we employed a lipid nanoparticle (LNP) system consisting of an ionizable lipid, a phospholipid, cholesterol, and a PEGylated lipid (Figure 7A) because the Lipofectamine-based lipoplex system used in *in vitro* experiments has poor nucleic acid encapsulation and thus low nuclease tolerability *in vivo*.^40^^,^^41^ We examined the transfection potency of LNP-encapsulated miR-145S1 into BiPL cells. We intravesically administered FITC-labeled miR-145S1-LNPs (20 μM solution in 100 μL of PBS, a total of 4.5 nmol/infusion) or PBS to the rat BiPL model established by the free intake of water containing 0.05% BBN and 0.005% Tween 80 for 16 weeks (Figure S10A). To prevent the early discharge of materials from the bladder, we sutured the external urethral orifice after administration, as indicated in Figures S10A and S10B. After an incubation for 3 h, the solution was released by the removal of the sutures. Animals were then sacrificed, and bladder tissue was collected, snap-frozen, cryosectioned, and mounted with the NucBlue nuclear stain for the direct detection of FITC signals. A confocal microscopic analysis revealed strong FITC signals in the cytoplasm and nuclei of urothelial cells (Figure S10C). We also performed miRNA ISH to detect the miR-145 molecule after administration. miR-145 signals were significantly increased in urothelial cells (Figure S10D), consistent with the results obtained by the direct detection of FITC. These results indicated that miR-145S1-LNPs were successfully delivered into BiPLs and that LNPs worked as a drug delivery system for intravesical administration.

### *In vivo* anti-tumor efficacy of miR-145S1-LNPs against premalignant lesions

We examined the anti-tumor efficacy of miR-145S1-LNP in the model using the multiple pathological and molecular indices examined above.

The rat BiPL model was established by the free intake of water containing 0.05% BBN and 0.005% Tween 80 for 16 weeks. After confirming the development of early lesions in the bladder in a few rats, we intravesically administered miR-145S1-LNPs or ctl-RNA-LNPs (20 μM of miRNA solution in 100 μL of PBS, total 4.5 nmol/infusion) and incubated the solution *in situ* for at least 3 h by suturing the external urethral orifice (Figure 7B). After twice weekly treatments for 2 weeks (a total of four treatments), animals were sacrificed 3 days after the final administration.

To assess the overall anti-tumor efficacy of the treatment, we evaluated the proliferative area using gross images and image analysis software (ImageJ). As described earlier, bladders were fixed *in situ*, sliced in the sagittal plane, and photographed on a black background with a macro lens-equipped camera (Figure 7C). In gross images, bladders had a thickened proliferative area that was white to pink in color, whereas the intact area was dark blue to black (Figure 7C). Therefore, we quantified the white and blue-black areas, which indicated affected and intact areas, respectively (Figure 7C). The results showed that the administration of miR-145S1-LNPs significantly decreased the proliferative area (Figures 7D–7F).

**Figure 8:**
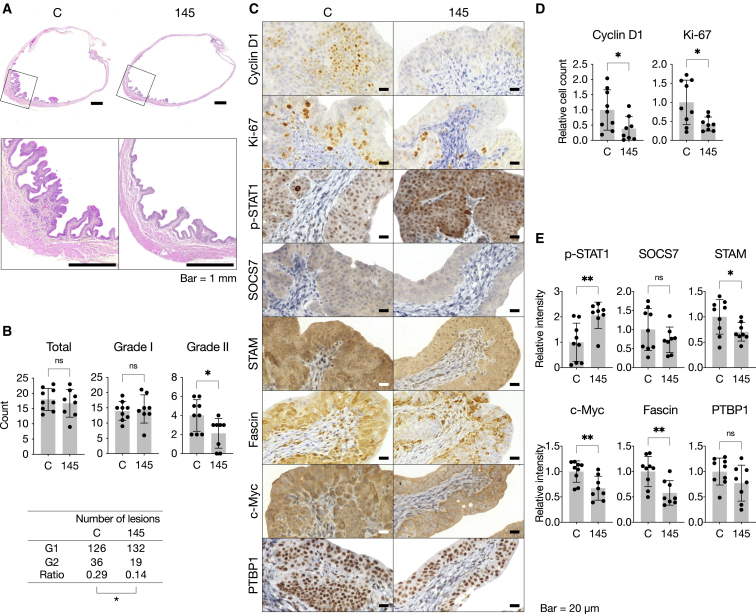
Effects of miR-145 replacement on histopathological and molecular markers (A) Representative loupe images of H&E-stained bladder tissues treated with miR-145S1-LNPs or ctl-RNA-LNPs. Note that the bladder treated with miR-145S1-LNPs had fewer and more minor lesions than that treated with Ctl-RNA-LNPs. Scale bars, 1 mm. (B) Number of lesions of each grade (grade I, G1; grade II, G2) in bladders treated with miR-145S1-LNPs (n = 8) or ctl-RNA-LNPs (n = 9). ∗p < 0.05; two-tailed unpaired Student’s t test for the count comparison; two-tailed Fisher’s exact test for the ratio comparison. Data are presented as means ± SD. (C) Representative images of immunohistochemical staining for disease-associated markers (cyclin D1, Ki-67, p-STAT1, SOCS7, STAM, Fascin, c-Myc, and PTBP1) in BiPLs treated with miR-145S1-LNPs (n = 8) or ctl-RNA-LNPs (n = 9). Scale bars, 20 μm. (D) Relative counts of cyclin D1- and Ki-67-positive cells in BiPLs treated with miR-145S1-LNPs (n = 8) or ctl-RNA-LNPs (n = 9). (E) Relative staining intensities of p-STAT1, SOCS7, STAM, c-Myc, Fascin, and PTBP1 in BiPLs treated with miR-145S1-LNPs (n = 8) or ctl-RNA-LNPs (n = 9). Data are presented as the means ± SD. ∗p < 0.05, ∗∗p < 0.01; ns, not significant; two-tailed unpaired Student’s t test unless otherwise indicated.

A histopathological examination revealed that most lesions in the treated group were grade I, with few grade II lesions being observed (Figures 8A and 8B). The ratio of grade II/grade I lesions was significantly decreased in the treatment group (ratio, 0.14; 19/132) compared with the control group (ratio, 0.29; 36/126; Figure 8B), while the total number of lesions did not significantly differ between the groups (treated, 151; control, 162; Figure 8B). Lesions in the miR-145S1-treated group rarely had mitotic figures, whereas the control group sporadically exhibited multiple areas with a relatively high mitotic index.

The IHC analysis showed that the treated group had significantly fewer cyclin D1-positive lesions than the control group (Figures 8C and 8D). Given that cyclin D1 could serve as the earliest marker of disease progression, this result suggested that miR-145 replacement inhibited disease progression at the early stage of tumorigenesis in the rat BiPL model. Similar to the results obtained for cyclin D1, the treated group had fewer Ki-67-positive lesions (Figures 8C and 8D), suggesting that the treatment inhibited the growth of BiPLs. In addition, the administration of miR-145S1 induced the downregulation of miR-145 target genes (Fascin, c-Myc, and STAM) and an increase in the level of a marker of IFN pathway activation (p-STAT1, Figure 8E). The expression of PTBP1 and SOCS7 was slightly downregulated in the miR-145-treated group, but this downregulation was not significant due to the high statistical dispersion (Figure 8E). These results suggested that the miR-145S1-LNP treatment inhibited the growth and disease progression of premalignant lesions.

The TUNEL assay showed that the number of apoptotic cells did not significantly differ among the groups examined (Figure S11A). Moreover, no massive necrosis or destruction of tissues was observed throughout the control and treated samples. The infiltration of lymphocytes and macrophages was mainly observed in the lamina propria of the affected areas, particularly in areas close to grade II lesions; however, the degree of inflammation was slight and did not markedly differ between the treated and control groups. No signs of bacterial infection secondary to the administration of LNP were observed throughout samples.

To assess the systemic effects of intravesically infused miR-145S1-LNP, we utilized miRNA qRT-PCR to evaluate the leakage of miR-145S1-LNP into the bloodstream. The results showed no significant increases in the plasma levels of miR-145 in the samples from rats receiving intravesical administration of miR-145S1-LNP (Figure S11B), indicating that the administered solution was successfully retained in the bladder and did not leak into the bloodstream. Throughout these experiments, the intravesical infusion of miR-145S1-LNP did not cause any significant systemic adverse effects, including behavioral disorders, moribund conditions, or death associated with the intravesical administration, and no recognizable gross lesions were detected in major organs, such as the heart, lungs, liver, kidneys, and spleen, during autopsy. Therefore, these results suggested that the systemic effects of the intravesical infusion of miR-145S1-LNP on the body were minimal.

## Discussion

Despite its pivotal necessity, the development of therapies for progressive NMIBC has been challenging due to the lack of validated *in vivo* models and therapeutic modalities that selectively target genetic vulnerability. We herein validated the molecular basis of rat BiPLs by clarifying their detailed profiles of somatic mutation, histopathological, and miR-145- and BC-related oncogene expression. These profiles highlighted the high molecular similarities between rat BiPLs and UROMOL2021 class 2a human NMIBC,^3^ indicating the suitability of the rat BiPL model as a model of human progressive NMIBC with a poor prognosis. We found that the downregulation of miR-145 appeared to be one of the earliest oncogenic events and demonstrated that miR-145 is the principal regulator of the premalignant BC phenotype characterized by downregulated IFN-STAT1/2 signaling and upregulated BC-related oncogenes, including c-Myc, Fascin, and PTBP1. Furthermore, we developed a novel miR-145-based therapeutic agent (miR-145S1) and demonstrated its efficacy against premalignant lesions *in vivo*. The present results provide insights into the essential roles of miR-145 in bladder tumorigenesis, as well as a validated interventional model and novel miRNA-based therapeutic modality targeting the molecular vulnerability of progressive NMIBC.

Rat BiPLs showed molecular signatures that were highly similar to those of human progressive NMIBC, particularly the UROMOL2021 class 2a subclass, which has the worst prognosis among NMIBCs.^3^ The expression profile of BiPLs at 18 weeks was cyclin D1+, cyclin A2+, and p53−, with the subsequent expression of p53 and increases in cyclin A2 after an additional 4 weeks. In addition, BiPLs harbored somatic mutations in genes such as *Birc6*, *Dync1h1*, *Pik3ca*, *Mycbp2*, *Atm*, and *Crebbp,* as well as SBS mutation signatures associated with APOBEC and cigarette smoke-related BC. These molecular profiles of BiPL were highly similar to the profiles of class 2a human NMIBC (late cell cycle marker, cyclin A2; p53 anomaly; low IFN; c-Myc expression; APOBEC mutation signatures).^3^ Although rat BiPLs had a slight mixture of the characteristics of classes 1 and 3 (the expression of the early cell cycle marker, cyclin D1), their overall phenotypes were distinct from those of the NMIBC subclasses with a favorable prognosis, including class 1 (represented by FGFR3 and Ras mutations), class 2b (represented by high IFN signaling), and class 3 (represented by FGFR3 mutations and RNA-editing mutation signatures),^3^^,^^33^ emphasizing the similarities between rat BiPLs and human progressive NMIBC with a poor prognosis. Furthermore, we identified mutations in *Pik3ca*, a frequently mutated oncogene in human NMIBC, with the subsequent upregulation of Akt phosphorylation in BiPL tissues. The mutation point was close to the hotspots of activating *PIK3CA* mutations in humans,^38^ which is consistent with the activation of PI3K/Akt in human NMIBC.^3^ These findings emphasize the high homology between the malignant progression sequences of rat BiPLs and human class 2a progressive NMIBC.

The primary molecular phenotype of rat BiPLs was associated with the suppression of IFN-STAT1/2 pathways or high c-Myc expression. Similar anomalies were observed in human BC, such as the frequent homozygous deletion/inactivation of α-IFN,^42^ c-Myc signatures in human NMIBC profiles,^33^ and c-Myc-mediated malignant progression in early human BC.^43^ The low IFN and high c-Myc signatures of rat BiPLs were consistent with those of human progressive NMIBC (class 2a, poor prognosis), and the low IFN profile was the key signature that distinguished class 2a (a poor prognosis) from other benign-oriented subclasses (classes 1, 2b, and 3).^3^ These findings further emphasize the similarities in molecular signatures between rat BiPL and human progressive NMIBC with a poor prognosis. These results also strongly suggest that IFN-STAT1/2- and c-Myc-associated signaling alterations shape the molecular basis for the progressive/premalignant characteristics of rat BiPLs and human progressive NMIBC.

Notably, the IFN-STAT1/2- and c-Myc-associated premalignant phenotypes were dominantly regulated by miR-145. The expression levels of IFN-STAT1/2-associated markers and c-Myc showed strong negative correlations with miR-145 expression both *in vitro* and *in vivo*. Transcriptome and immunoblot analyses demonstrated that miR-145 replacement induced the strong and dominant activation of IFN-STAT1/2 signaling and significant downregulation of c-Myc expression in BC cells, likely by both direct and IFN-STAT1/2-mediated indirect mechanisms. These results were further confirmed by *in vivo* miR-145 replacement therapy significantly inhibiting disease progression with the activation of the IFN-STAT1/2 pathway and suppression of the c-Myc pathway in the rat BiPL model. These findings strongly suggest that miR-145 is the principal regulator of class 2a-like progressive characteristics in early BC, thereby rationalizing the targeting of miR-145 downregulation as a therapeutic approach for early BC.

miR-145S1-based intravesical infusion therapy successfully inhibited the progression of premalignant lesions *in vivo*. The advantages of miR-145S1-based replacement therapy are as follows. First, miR-145S1 potently, selectively, and simultaneously targets a wide range of essential oncogenes for bladder tumorigenesis; thus, it is likely to have higher efficacy than non-specific (BCG) or single-molecule targeting agents (siRNAs or small-molecule inhibitors). Second, intravesically administered miR-145S1 directly reaches and affects premalignant lesions localized in the mucosal layer despite its lower vascularization and thus would be more effective than systemic therapeutic agents. Third, miR-145 is a non-bacterial agent with safer properties than BCG immunotherapy and no concerns of systemic infection, as observed with the BCG immunotherapy. In addition, this strategy employs local administration, which minimizes the reported drawbacks of miRNA-based therapy, such as the hepatic accumulation of administered agents^44^ and toxicities from systemic immunological adverse events.^45^ These features imply the high potential of miR-145S1 replacement as a new intravesical therapeutic modality for premalignant BC lesions.

The present results revealed that miR-145 replacement therapy had a feature of RNAi-mediated IFN activation therapy. Based on the results obtained herein, the activation mechanism appears to be based on RNAi against the IFN signaling inhibitors SOCS7 and STAM, which is consistent with previous findings.^29^ IFN therapy has been evaluated as BC therapy due to its cytotoxic and anti-angiogenic activities.^46^^,^^47^ The intravesical instillation of rhIFNα was unsuccessful, which may have been due to the short half-life of the protein.^48^ However, the continuous overexpression of IFN by adenoviral transduction (rAd-IFN/Syn-3, also called Nadofaragene Firadenovec) showed significant efficacy and safety *in vivo*^49^ and in clinical trials, including phase I (43% CR at 3 months, NCT01162785),^50^ phase II (no recurrence for 12 months in 35% of high-grade BCG refractory or relapsed NMIBC patients, NCT01687244),^51^ and phase III (CR for 3 months in 53.4% of patients with BCG-unresponsive CIS; CR for 12 months in 45.5% of CIS patients, NCT02773849) trials.^52^ These findings highlight the strong potential of miR-145-mediated IFN activation to be therapeutically effective against premalignant lesions with or without BCG resistance. Furthermore, given that miR-145 utilizes a unique mechanism to activate IFN signaling, miR-145 replacement may be synergetically effective with conventional therapies that use IFN ligands or BCG.

In the assessment of the *in vivo* delivery of miRNA-LNP, FITC or ISH signals indicating the distribution of miR-145 molecules were the most intense on the mucosal surface and gradually decreased toward the deeper layer of the mucosa. Fluorescent microscopy or ISH detected only weak or no signals in the deeper layer of the mucosa; however, the target proteins of miR-145 were significantly downregulated, even in the deep layer without FITC/ISH signals. This inconsistency appears to be due to the limited sensitivity and dynamic ranges of fluorescent or ISH-mediated detection, which cannot identify a small number of miRNA molecules in cells or tissues. Given that miRNA molecules are known to efficiently induce RNAi because of their RISC-involved catalytic reactions,^7^ a small number of miR-145 molecules undetectable by ISH or fluorescent microscopy may induce the downregulation of the target genes. Indeed, the major target proteins of miR-145, such as Fascin and c-Myc, had an exact opposite expression pattern from that of the miR-145 signal distribution in samples from rats receiving miR-145 replacement, i.e., the degree of target gene downregulation was the most intense on the surface layer, and its magnitude gradually decreased toward the deeper layer of the mucosa. These results support the hypothesis that miR-145S1 reached into the deeper layer of mucosa with levels that were undetectable by ISH or fluorescent microscopy and exerted RNAi functions; however, further studies to validate these results are needed in the future.

As a limitation, rat BiPLs had different morphological characteristics from those of CIS, a major histological subtype of human progressive NMIBC. The major histological subtype of rat BiPLs was PN hyperplasia (papillary growth), which differs from the main histological subtype of CIS (flat growth). However, despite these histological differences, the rat BiPL model still has high potential as a molecular-based model of human progressive NMIBC. Rat BiPLs have a strong premalignant predisposition with a high progression rate along with *Tp53* mutations,^23^ whereas the progressive characteristics of BiPLs are different from the morphologically equivalent subtype in humans (human papillary hyperplasia), which has a benign-oriented disease course.^21^^,^^23^^,^^53^ These findings are consistent with recent studies demonstrating that the progressive characteristics of NMIBC (recurrence rate, progression rate, and prognosis) are defined by molecular characteristics rather than the histological subtype.^3^ Indeed, UROMOL2021 class 2a, which includes many Ta tumors along with CIS, is strongly associated with the worst prognosis among NMIBCs and has a high recurrence rate.^3^ In addition, BBN-induced PN hyperplasia that developed in rats exhibited neither somatic mutations nor the overexpression of FGFR3, a benign-oriented marker frequently mutated/overexpressed in human NMIBC.^32^ Given that FGFR3 expression is absent in class 2a progressive NMIBC and CIS,^3^^,^^54^ these results further support the hypothesis that the FGFR3-negative rat BiPL model has premalignant molecular features similar to those of progressive NMIBC despite morphological differences. Nevertheless, miR-145 suppressed FGFR3 as a direct target; thus, regardless of the FGFR3 mutation/expression status, miR-145-based therapy may be effective against a broad spectrum of NMIBCs, including different UROMOL2021 classes (classes 1, 2a/b, and 3) and TNM subtypes (Ta and CIS), via the combined inhibition of c-Myc/cyclin D1, FGFR3, and the activation of IFN signaling.

miR-145S1 exerted slightly different effects from miR-145WT in the regulation of its direct targets, such as Fascin, although the target spectrum of miR-145S1 was largely similar to that of miR-145WT. In miRNA target regulation, the guide strand and its seed sequences are the most crucial factors to determine the target genes for each miRNA.^7^ Although miR-145S1 has identical seed sequences to those of miR-145WT in the guide strand, the passenger strand of miR-145S1 is modified to fully match its guide strand and thus differs from miR-145WT. These differences in the passenger strand may affect its affinity to the target genes and result in the minor difference between miR-145S1 and miR-145WT on Fascin. However, it is important to note that miR-145S1 could also downregulate Fascin expression more strongly in the *in vivo* context than in the *in vitro* context, suggesting that miR-145S1 also has the capability to strongly downregulate Fascin expression depending on the microenvironment. Nevertheless, miR-145S1 showed a similar spectrum for the target gene regulation to miR-145WT. Therefore, regardless of affinity differences in different contexts, miR-145S1 may serve as a therapeutic modality for the replacement of miR-145 expression in early BC.

## Materials and methods

### Cell lines and culture

T24, JBV, NBT-T1, and NBT-L2B cells were cultured in EMEM (Wako, Osaka, Japan) supplemented with 10% FBS (Sigma-Aldrich, St. Louis, MO). HBlEpC and RNM cells were cultured in HBlEpC Growth Medium (Cell Applications, San Diego, CA, 217K-500). T24 cells were obtained from the Japanese Cancer Research Resources Bank, NBT-T1 and NBT-L2b cells were obtained from the Riken Bioresource Research Center, 253J-BV cells were obtained from Osaka Medical College, and HBlEpC cells were obtained from Cell Applications. RNM cells were established as primary cultured cells from rat bladder urothelium using a previously reported method^55^ and were confirmed to be of epithelial origin by an immunoblot analysis of pan-CK expression. The cell lines used were confirmed to be negative for mycoplasma by mycoplasma testing (MycoAlert, Lonza, Basel, Switzerland). All cells were cultured at 37°C in a humidified incubator with 5% CO_2_. Cell viability was assessed using the trypan blue exclusion test.

### miRNA synthesis and miRNA/siRNA transfection

miR-145WT was purchased from Thermo Fisher Scientific, Waltham, CA (miR-145-5p mirVana miRNA mimic, MC11480). miR-145S1 and other miR-145 derivatives were designed by the authors and synthesized by Hokkaido System Science (Hokkaido, Japan). siRNAs for human SOCS7/STAM and rat Socs7/Stam were purchased from Sigma-Aldrich. Non-targeting control miRNA sequences with proven suitability in previous studies^13^^,^^18^ were synthesized by Hokkaido System Science and used as negative control sequences (control miRNA).

In transfection experiments, cells were seeded on six-well plates at a concentration of 0.2 × 10^5^ cells/mL. Cells were transfected with a mixture of nucleic acids (20 nM for miRNAs and 10 nM for siRNAs) and Lipofectamine RNAiMAX (Thermo Fisher Scientific). After an incubation for 48 or 72 h, effects were examined using each method.

### qRT-PCR analysis

Total RNA was extracted and purified using the NucleoSpin miRNA Kit (MACHEREY-NAGEL, Düren, Germany). RNA concentrations and integrity were examined using UV spectrophotometry and the Agilent 2100 Bioanalyzer (Agilent Technologies, CA), respectively. To measure miRNA, total RNA (0.5 μg) was reverse transcribed to cDNA using the TaqMan MicroRNA Reverse Transcription Kit (Thermo Fisher Scientific). cDNA was amplified by PCR with the THUNDERBIRD Probe qPCR Mix (TOYOBO, Osaka, Japan, QPS-101) and TaqMan MicroRNA Assays (Thermo Fisher Scientific) to evaluate the expression of miR-145 (assay ID 002278), RNU6B (assay ID 001093), RNU19 (assay ID 001003), and RNU48 (assay ID 001006). To measure mRNA, total RNA (0.5 μg) was reverse transcribed to cDNA using the PrimeScript RT Reagent Kit (Takara, Shiga, Japan). cDNA was amplified by PCR with the THUNDERBIRD SYBR qPCR Mix (TOYOBO, QPS-201) with gene-specific primers (see Table S1). Amplified fluorescent signals were recorded in each cycle using the TaKaRa Thermal Cycler Dice Real-Time System II (Takara). The relative expression of each miRNA or mRNA was calculated using the ΔCt method with normalization to RNU6B (for miRNAs) or TBP (for mRNAs). The internal control was selected from the candidate genes (RNU6B, RNU19, and RNNU48 for miRNAs; ACTB, GAPDH, and TBP for mRNAs) using NormFinder (Aarhus University Hospital, Denmark). All PCRs were performed in triplicate.

### Northern blotting for miRNA

Total RNA was extracted and purified using the NucleoSpin miRNA Kit (MACHEREY-NAGEL). RNA concentrations and integrity were examined using UV spectrophotometry and the Agilent 2100 Bioanalyzer (Agilent Technologies), respectively. Total RNA (5 μg) was mixed with 2× RNA loading buffer without ethidium bromide (Wako, 182-02571) and incubated at 70°C for 10 min. After a pre-run at 300 V for 1 h, the RNA samples and an RNA ladder marker (DynaMarker DIG Labeled Blue Color Marker for Small RNA, BioDynamics Laboratory, Tokyo, Japan, DM270) were electrophoresed at 200 V for 50 min on a 15% polyacrylamide gel with 4 M urea (SuperSepTMRNA 15%, Wako, 194-15881) and RNase-free Tris-borate-EDTA buffer (TBE buffer) (Wako, 318-90041). All experimental equipment was cleaned with RNaseZap (RNaseZap RNase Decontamination Wipes, Thermo Fisher Scientific, AM9786,) to remove RNase. The electrophoresed gel was washed for 10 min with TBE buffer, and RNA in the gel was transferred onto a nylon membrane (BrightStar-Plus Positively Charged Nylon Membrane, 15 × 15 cm, Thermo Fisher Scientific, AM10100) at 15 V for 40 min with a Trans-Blot SD Semi-Dry Transfer Cell (Bio-Rad). The transferred membrane was rinsed with 0.2× SSC for 1 min and baked at 80°C for 2 h in an oven. The membrane was pre-hybridized at 53°C for 30 min with 10 mL of hybridization buffer (ULTRAhyb Ultrasensitive Hybridization Buffer, Thermo Fisher Scientific, AM8670) and then hybridized at 53°C for 1 h with 0.4 nM of the digoxigenin-labeled miR-145 probe (QIAGEN, Hilden, Germany, no. 339456). After three washes for 15 min with 5× SCC, the membrane was blocked for 1 h with the PVDF Blocking Reagent for Can Get Signal (TOYOBO, NYPBR01). Subsequent to a wash with TBST for 15 min, the membrane was reacted for 1 h with an anti-digoxigenin antibody conjugated with peroxidase in Can Get Signal Immunoreaction Enhancer Solution (final concentration [1:10,000], TOYOBO, NKB-101T). After three washes with TBST for 5 min, membranes were incubated with Luminata Forte Western HRP substrate (EMD Millipore) to visualize the signals.

### cDNA microarray analysis

Total RNA was extracted using the Maxwell RSC simplyRNA Cells Kit (Promega, Madison, WI, AS1390) and a Maxwell RSC instrument (Promega, AS4500). BC cells (253 JB-V) were transfected and incubated for 48 h with miRNAs (miR-145-5p, 10 nM; control miRNA, 10 nM) and Lipofectamine RNAiMAX (Thermo Fisher Scientific). After two PBS washes, cells were lysed in homogenization solution (400 μL) with 1-thioglycerol (4 μL). Subsequently, the lysate (200 μL) was mixed with lysis buffer (200 μL), vortexed for 15 s, and transferred to a Maxwell RSC Cartridge. After an incubation with DNase I, the cartridge was inserted into the Promega Maxwell RSC instrument to purify total RNA. RNA integrity was assessed using an Agilent 2100 Bioanalyzer (Agilent). RNA samples were labeled with the Agilent Low Input Amp Labeling Kit (Agilent, cat. no. 5190-2305) and hybridized on slides of SurePrint G3 Human GE Microarray 8x60K version 3.0 (Agilent) in the Agilent G2545A Hybridization Oven (Agilent) at 65°C overnight. Slides were then scanned with the Agilent Microarray Scanner System (Agilent, G2565CA). Signals were normalized to the 75th percentile of the signal intensity by utilizing the Subio platform version 1.2.2 (Subio, Kagoshima, Japan). Normalized signals were log2 transformed and used to calculate FC values for comparisons of gene expression in miR-145- and control miRNA-transfected cells. p values were calculated by the Student’s t test (paired, two-tailed) from two biological replicates. Genes with an absolute FC of greater than 1.5 and p values of less than 0.05 were defined as DEGs. DEGs were then subjected to a Metascape analysis using the Kyoto Encyclopedia of Genes and Genomes and Reactome pathway databases.^56^ JMP version 12.2 (SAS Institute, Cary, NC) was used to generate heatmaps. A circos plot was generated using a function of Metascape.^56^

### Immunoblotting

In the immunoblot analysis, cells and tissues were lysed with 1% SDS buffer. In brief, cultured cells were fixed for 30 min with 10% TCA after two washes with ice-cold PBS (Wako). Subsequent to two washes with ice-cold PBS, fixed cells were lysed with 1% SDS buffer (Wako). Tissues were snap-frozen using liquid nitrogen and then homogenized in 1% SDS buffer (Wako). Whole-cell lysates were sonicated and centrifuged at 13,000 × *g* for 20 min. Protein concentrations in the supernatants were measured with a DC Protein Assay Kit (Bio-Rad, Hercules, CA). Protein samples (1–5 μg/lane) were subjected to 10%–12.5% SDS-PAGE and transferred to polyvinylidene fluoride membranes with a pore size of 0.45 μm (Immobilon-P Membrane, EMD Millipore, Burlington, MA). After 1 h of blocking with PVDF Blocking Reagent (TOYOBO), membranes were incubated with primary antibodies at 4°C overnight. The primary antibodies used for immunoblotting are summarized in Table S2. After three washes with TBS-T for 5 min each, membranes were incubated at room temperature with horseradish peroxidase (HRP)-conjugated anti-mouse or anti-rabbit secondary antibodies (1:4,000 dilution, Cell Signaling Technology, Danvers, MA). After three TBS-T washes, membranes were incubated with Luminata Forte Western HRP substrate (EMD Millipore) to visualize immunoreactions. Sample loading was checked with an anti-α-tubulin antibody (MBL, Tokyo, Japan, PM054).

### Animal experiments

All animal experiments were approved by the Institutional Animal Care and Use Committee (IACUC) of Gifu University. All animals were housed with ad libitum-access to food and water under the standard light-dark cycle.

The BiPL model for gross, histological, and molecular assessments was established in F344/NSIc rats (n = 8, female, 4 weeks old; SLC, Shizuoka, Japan) by the free intake of water containing 0.05% BBN (Tokyo Chemical Industry, Tokyo, Japan) and 0.005% Tween 80 (Sigma-Aldrich) for 16 weeks. BBN exposure was terminated at 16 weeks, and rats were housed with normal distilled water until weeks 18 or 22 and then sacrificed for subsequent analyses. To consistently evaluate premalignant lesions, bladder samples for gross, hematoxylin and eosin (H&E), and single IHC evaluations were fixed *in situ* by a uniform infusion of 300 μL of 4% paraformaldehyde into the bladder and were then sliced in the sagittal plane. In miRNA ISH and ISH-IHC double-staining analyses, bladder samples were embedded in Tissue-Tek OCT Compound (Sakura Finetek Japan, Tokyo, Japan) and snap-frozen using liquid nitrogen. In miRNA qRT-PCR and NGS analyses, tissue samples were directly snap-frozen in liquid nitrogen and used for analyses.

In miR-145S1 delivery experiments, an LNP system was employed since the Lipofectamine-based lipoplex system used in *in vitro* experiments has poor nucleic acid encapsulation and thus low nuclease tolerability *in vivo*.^41^ miR-145S1 conjugated to FITC was encapsulated in LNPs consisting of an ionizable lipid, a phospholipid, cholesterol, and PEGylated lipid using the NanoAssemblr system (Precision NanoSystems, Vancouver, Canada).^40^ To test transfection potency *in vivo*, FITC-labeled miR-145S1-LNPs (20 μM solution in 100 μL of PBS, a total of 4.5 nmol/infusion) or PBS was intravesically administered to rats with BiPLs developed over 16 weeks. The dose of miRNA-LNPs was determined to be 4.5 nmol/infusion based on preliminary experiments that test the transfection potency of miRNA-LNPs at doses of 2.25, 4.5, and 9 nmol/infusion. The external urethral orifice was sutured after administration to prevent the early discharge of materials from the bladder. After 3 h, the solution was released by the removal of sutures. Animals were then sacrificed, and bladder tissue was collected, snap-frozen in Tissue-Tek OCT Compound (Sakura Finetek Japan), cryosectioned, and mounted with the NucBlue nuclear stain for the direct detection of FITC signals with confocal microscopy. Frozen sections were also used to detect miR-145 molecules by miRNA ISH.

In the experiment testing the therapeutic efficacy of miR-145S1, the BiPL model was established by the free intake of water containing 0.05% BBN and 0.005% Tween 80 for 16 weeks. After confirming the development of early lesions in the bladder in a few rats, miR-145S1-LNPs or ctl-RNA-LNPs (20 μM miRNA solution in 100 μL of PBS, a total of 4.5 nmol/infusion) were intravesically administered and incubated *in situ* for at least 3 h by suturing the external urethral orifice. After a twice weekly treatment for 2 weeks (a total of four treatments), rats were sacrificed 3 days after the final administration.

### Gross image analysis

In the assessment of overall anti-tumor efficacy, the areas of proliferative lesions were evaluated using high-resolution gross images and image analysis software. As described earlier, bladders were fixed *in situ*, sliced in the sagittal plane, and photographed with a macro lens-equipped camera (M.Zuiko Digital ED 60 mm F2.8 Macro and E-PL9 Mirrorless Camera, Olympus, Tokyo, Japan) on a black background. In gross images, the area with hyperplastic lesions had a thickened urothelial layer that was white to pink in color, whereas the normal area was dark blue to black. Therefore, blue-black and white areas were marked and quantified using ImageJ (NIH, Bethesda, MD) as intact and affected areas, respectively.

### Histology, IHC, and miRNA ISH

In H&E staining, IHC staining, and TUNEL assays, tissues were fixed with 4% neutral-buffered paraformaldehyde and embedded in paraffin. Paraffin-embedded samples were sectioned at a thickness of 5 μm and mounted on glass slides (S2226 for H&E staining, SCRE-05 for IHC staining, Matsunami Glass, Osaka, Japan). Sections were deparaffinized with Lemosol and rehydrated in graded alcohol series. H&E staining was performed with Tissue-Tek Hematoxylin 3G (Sakura Finetek Japan, 9131-4P) and Tissue-Tek Eosin (Sakura Finetek Japan, 8659). A histopathological diagnosis was performed by pathologists certified by the Japanese College of Veterinary Pathologists (JCVP) or the American College of Veterinary Pathologists (ACVP). In IHC staining, sections were then autoclaved at 120°C for 1 min in antigen retrieval buffer (ImmunoActive IA6500, Matsunami Glass), cooled to room temperature, and incubated with 0.3% hydrogen peroxide in methanol for 20 min. After blocking with 2.5% normal horse serum (Vector Laboratories, Newark, CA, S-2012-50), sections were incubated with primary antibodies at 4°C overnight. The primary antibodies used are listed in Table S2. After three washes with TBS-T, sections were incubated for 20 min with an HRP-conjugated secondary antibody (ImmPRESS HRP Horse Anti-Rabbit IgG Polymer Detection Kit, Vector Laboratories, MP-7401). The sections were washed three times with TBS-T, developed with DAB chromogen solution (ImmPACT DAB Substrate, Vector Laboratories, SK-4105), and counterstained with hematoxylin. Regarding TUNEL, sections were incubated with Proteinase K antigen retrieval solution, incubated for 20 min in 0.3% hydrogen peroxide in methanol, and labeled with TdT enzyme and FITC (TUNEL Assay Kit, Abcam, Cambridge, UK, ab206386). After blocking with normal serum, sections were incubated with the HRP-conjugated anti-FITC antibody. Signals were visualized with DAB chromogen solution, and sections were counterstained with hematoxylin. Histopathological images were acquired with a microscope (KEYENCE, Osaka, Japan, BZ-X700).

Single miRNA ISH was performed with hsa-miR-145-5p miRCURY LNA miRNA ISH Optimization Kits (QIAGEN, no. 339456). All solutions used in the experiment were prepared under RNase-free conditions. Tissues were embedded in Tissue-Tek OCT Compound (Sakura Finetek Japan), snap-frozen in liquid nitrogen, sectioned at a thickness of 10 μm with a cryostat (Leica Biosystems, Wetzlar, Germany, CM1860), mounted on glass slides (Matsunami Glass, PRO-01), and dried at 37°C overnight. Subsequently, sections were fixed with 4% neutral-buffered paraformaldehyde for 20 min, washed with PBS four times, and incubated with 50 μL of miRNA hybridization mix at 51°C for 50 min in a Dako hybridizer (Dako, Agilent Technologies). The miRNA hybridization mix included 25 μM miRNA probes (0.8 μL/slide) and ISH buffer (50 μL/slide, 1:625 dilution, final concentration [40 nM], denatured at 90°C for 4 min). Sections were sequentially washed (51°C, 5 min, 3 times) with 0.2× saline sodium citrate buffer (UltraPure SSC 20×, Thermo Fisher Scientific, 15557-044), washed with PBS-T for 1 min, blocked for 10 min with 2.5% normal horse serum (Vector Laboratories, S-2012-50), and incubated at room temperature for 1.5 h with 100 μL of an anti-DIG-alkaline phosphatase (ALP) antibody (1:50, Roche, Basel, Switzerland, 11093274910). After three PBS-T washes for 3 min each, signals were visualized with NBT/BCIP solution (incubation at 30°C for 2 h; SIGMAFAST BCIP/NBT, Sigma-Aldrich, B5655-5TAB) supplemented with levamisole (final concentration [0.2 mM], Sigma-Aldrich, 31742-250MG). After signal visualization, the ALP reaction was terminated by two washes with KTBT buffer (a mixture of 50 mM Tris-HCl, 150 mM NaCl, and 10 mM KCl at the final concentration) for 5 min each. Slides were washed with distilled water twice for 1 min each, counterstained for 1 min with Nuclear Fast Red (Vector Laboratories, H-3403), washed for 10 min with distilled water, dehydrated by passages through graded alcohol series, cleared with Lemosol, and mounted with MOUNT-QUICK (Daido Sangyo, Tokyo, Japan). Histopathological images were acquired with a microscope (KEYENCE, BZ-X700) and analyzed with ImageJ (NIH).

In miRNA ISH-IHC double staining, ISH steps were performed with hsa-miR-145-5p miRCURY LNA miRNA ISH Optimization Kits (QIAGEN, no. 339456). All solutions used for the ISH steps were prepared under RNase-free conditions. Tissues were embedded in Tissue-Tek OCT Compound (Sakura Finetek Japan), snap-frozen in liquid nitrogen, sectioned at a thickness of 10 μm with a cryostat (Leica Biosystems, CM1860), mounted on glass slides (Matsunami Glass, PRO-01), and dried at 37°C overnight. Subsequently, sections were fixed with 4% neutral-buffered paraformaldehyde for 20 min, washed with PBS four times, and incubated with 3% hydrogen peroxide in methanol for 20 min. Slides were incubated with 50 μL of miRNA hybridization mix at 51°C for 50 min in a Dako hybridizer (Dako, Agilent Technologies). The miRNA hybridization mix included 25 μM miRNA probes (0.8 μL/slide) and ISH buffer (50 μL/slide, 1:625 dilution, final concentration [40 nM], denatured at 90°C for 4 min). Sections were washed with 0.2× saline sodium citrate buffer (51°C, 5 min, 3 times; UltraPure SSC 20×, Thermo Fisher Scientific, 15557-044), washed with PBS-T for 1 min, blocked for 10 min with 2.5% normal horse serum (Vector Laboratories, S-2012-50), and incubated at room temperature for 1.5 h with 100 μL of an anti-DIG-peroxidase antibody (1:50, Roche, 11207733910). After three PBS-T washes for 3 min each, signals were visualized with a cyanine 5-tyramide amplification system (TSA Cyanine 5 System, SKU NEL705A001KT, Akoya Biosciences, Marlborough, MA). Sections were washed with PBS-T for 3 min, incubated with primary antibodies at 4°C overnight (Table S2), washed with PBS-T three times for 3 min each, incubated for 2 h with an Alexa Fluor 488-conjugated secondary antibody (1:100, Cell Signaling Technology), washed with PBS-T three times for 5 min each, and then mounted with the ProLong Glass Antifade Mountant with NucBlue Stain (Thermo Fisher Scientific, P36981). Fluorescence images were acquired with a confocal laser scanning microscope (Carl Zeiss, Oberkochen, Germany, LSM-710) or a conventional fluorescence microscope (KEYENCE, BZ-X700) and analyzed with ImageJ (NIH).

### Whole-genome sequencing

Samples for whole-genome sequencing were obtained from the single rat tissues of BiPL (P1, P2, and P3) or the adjacent normal urothelium (N1) at 18 weeks. Genomic DNA (0.4 μg, 10 ng/μL) was extracted using the QIAamp DNA Mini Kit (QIAGEN), randomly fragmented by sonication, analyzed for quantity, integrity, and purity using the 5400 Fragment Analyzer System (Agilent), and used for 150 PE sequencing on the NovaSeq6000 platform (Illumina, San Diego, CA). Sequence data were aligned with a rat reference genome (mRatBN7.2/rn7 Nov. 2020) using the Burrows-Wheeler Aligner (version 0.7.17), SAMtools, and Picard. Somatic SNPs and indels were identified by GATK (gatk-4.2.5.0, Broad Institute of MIT, Cambridge, MA), ANNOVAR,^57^ and the rat SNP/indel database (The Rat Genome Database^58^). Somatic CNVs and SVs were identified using CNVkit^59^ and BreakPointer (Broad Institute of MIT),^60^ respectively. Mutation signatures were analyzed using Signal.^61^ BiPL-specific somatic variants were calculated by subtracting variants in the adjacent normal tissue (N1) from those in BiPL tissues (P1, P2, and P3).

### Study approval

All animal experiments were approved by the IACUC of Gifu University (approval no. 2020-57) and performed according to the NIH Guide for the Care and Use of Laboratory Animals (National Academies Press, 2011).

### Statistical analysis

GraphPad Prism 8 version 8.4.0 (GraphPad Software, San Diego, CA), JMP version 12.2 (SAS Institute), and CutoffFinder^62^ were used for statistical analyses and heatmap generation. Detailed information on error bars, p values, and statistical tests is given in the figure captions. Unless otherwise specified, all experiments were conducted with biological replicates (sample size, n = 3), and measurements were taken from distinct samples in each experiment. All experiments were performed at least twice, and data for technical and biological replicates were reliably reproduced.

## Data Availability

cDNA microarray data have been deposited in the Gene Expression Omnibus under accession code GSE214209. NGS data have been deposited in the DDBJ Sequence Read Archive under accession codes DRR411656-DRR411659. Data from TCGA microRNA data are available in the TCGA Research Network (https://www.cancer.gov/tcga).

## Supplemental information

Document S1. Figures S1–S11 and Tables S1 and S2

Document S2. Article plus supplemental information
