# Supplementary material for: Targeting microRNA-145-mediated progressive phenotypes of early bladder cancer in a molecularly defined in vivo model
Source: Mol Ther Nucleic Acids. 2023 Jul 3;33:960–82. doi: 10.1016/j.omtn.2023.06.009 (PMC10505924; doi:10.1016/j.omtn.2023.06.009)
Supplement: Document S1. Figures S1–S11 and Tables S1 and S2 [file mmc1.pdf]

OMTN, Volume *33*

Supplemental information

Targeting microRNA-145-mediated progressive phenotypes of early bladder cancer in a molecularly defined *in vivo* model

Kazuki Heishima, Nobuhiko Sugito, Chikara Abe, Akihiro Hirata, Hiroki Sakai, and Yukihiro Akao

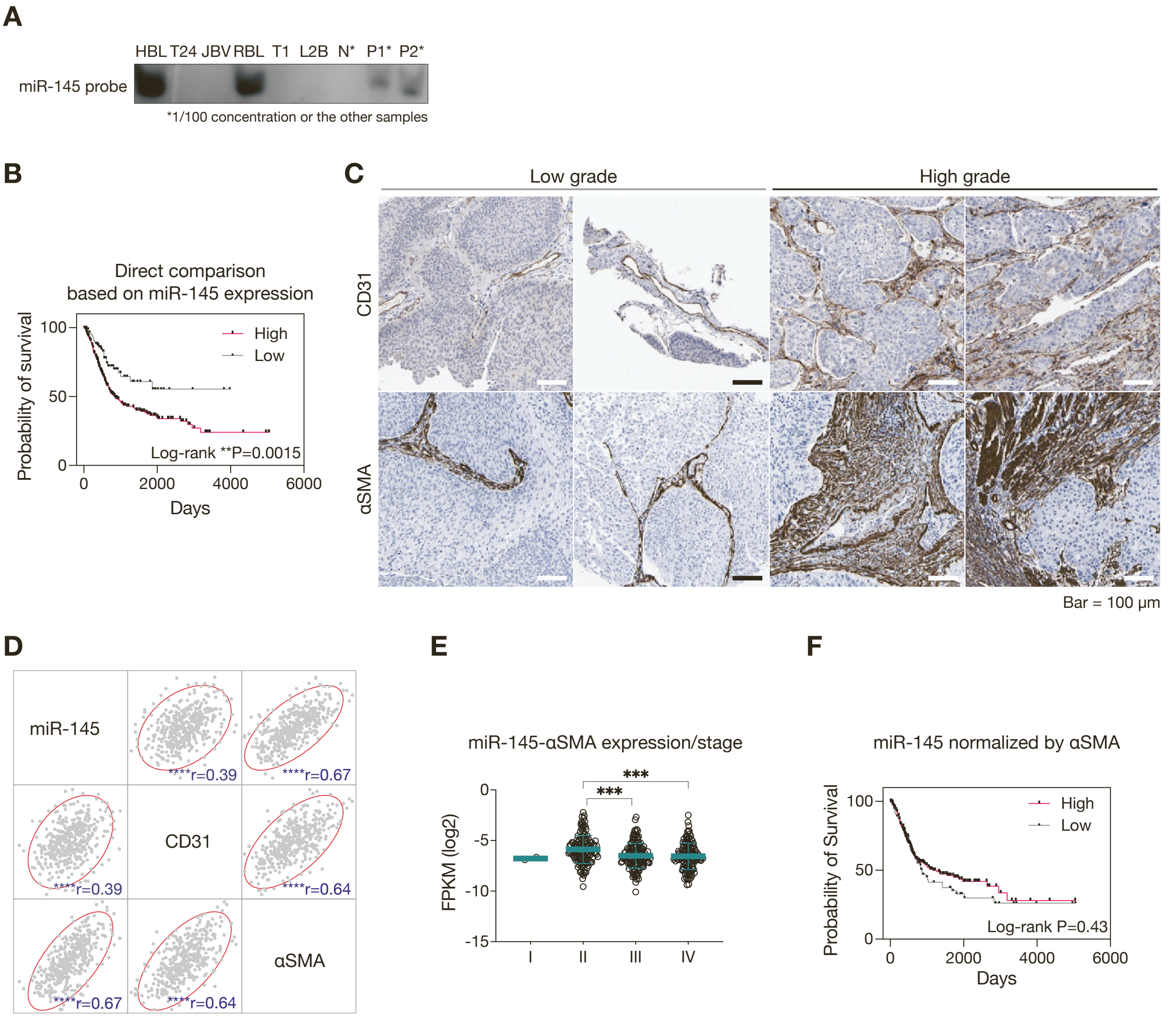

Figure S1

(**A**) Northern blotting for miR-145 expression in steady-state human and rat BC cell lines, as well as positive and negative controls utilizing T24 cells treated for 48 hours with 20 nM of control miRNA (N), miR-145WT (P1), and miR-145S1 (P2). Human BC cell lines, T24 and 253 JB-V (JBV); Rat BBN-induced BC cell lines, NBT-T1 (T1) and NBT-L2B (L2B); Normal diploid cells, HBlEpC (HBL, human) and RNM (rat, primary culture). Due to the high concentration of exogenous miR-145, total RNAs from T24 cells treated with 20 nM of control miRNA (N), miR-145WT (P1), and miR-145S1 (P2) were diluted to 1:100 before their application for Northern blotting. Blots were visualized with chemiluminescence using a digoxigenin-labeled miR-145 probe. (**B**) Kaplan-Meier survival curves for BC patients with high and low miR-145 expression levels. Survival data were obtained from the TCGA database. **P<0.01; the Log-rank test. (**C**) Representative images of high- and low-grade BC samples in the tissue microarray with immunohistochemistry staining for CD31 and αSMA. Note that high-grade BC samples contained more CD31-positive blood vessels and αSMA-positive smooth muscle than low-grade BC samples. Images were obtained from the Human Protein Atlas. Scale bars: 100 µm. (**D**) Correlation maps for miR-145, CD31, and αSMA expression. Expression data were obtained from the TCGA database. ****P<0.0001, Pearson's correlation coefficient. (**E**) miR-145 expression normalized to αSMA expression in each stage. Expression data were obtained from the TCGA database. ***P<0.001; a one-way ANOVA with Dunnett's post hoc test. Data are presented as means ± SDs. (**F**) Kaplan-Meier survival curves for patients with BC with high and low miR-145 expression normalized to αSMA expression. Survival data were obtained from the TCGA database. Survival associations were tested by the Log-rank test.

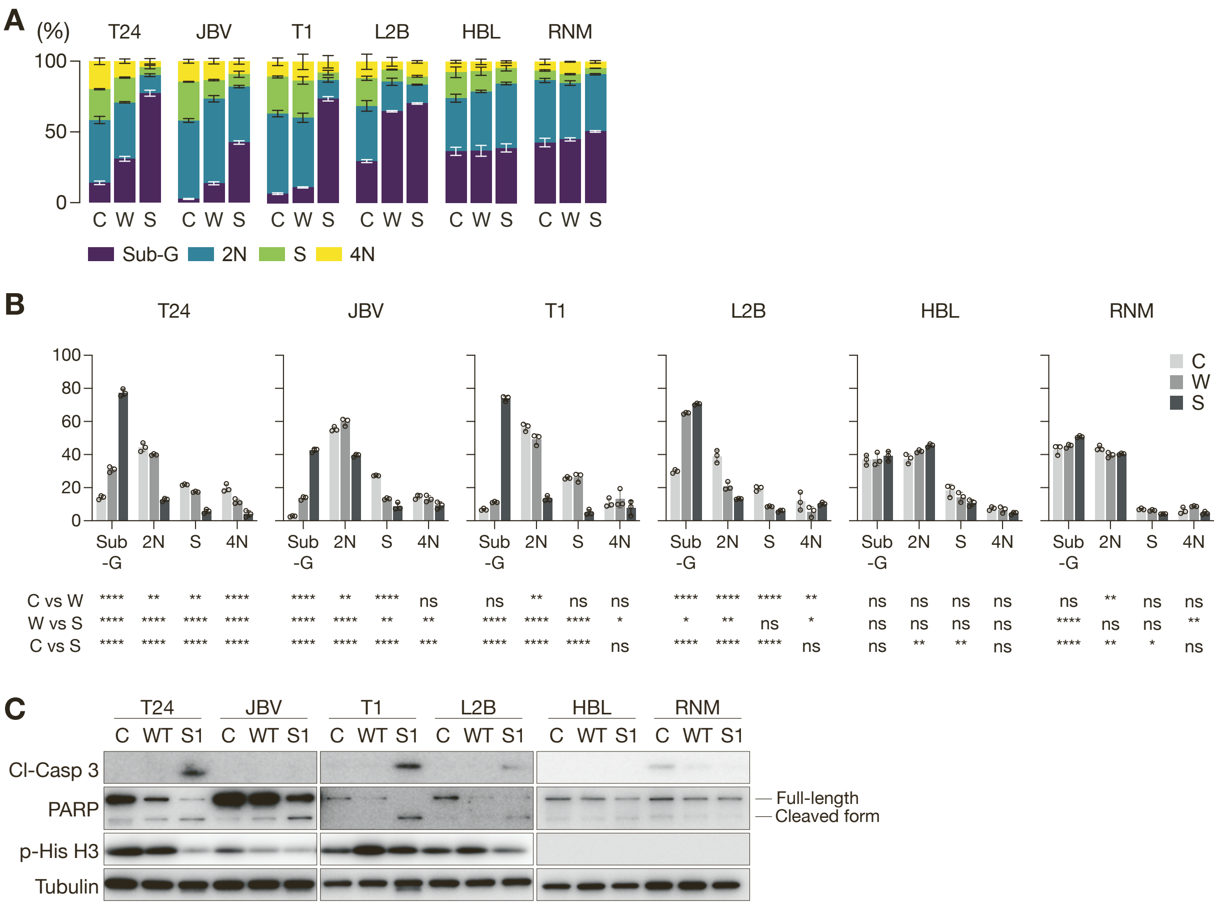

Figure S2

(**A**) Cell cycle analysis of human and rat BC cells treated with miR-145WT (W), miR-145S1 (S), or control miRNA (C) at 20 nM for 72 hours. (**B**) Statistical analysis of cell cycle data in human and rat BC cells treated with miR-145WT (W), miR-145S1 (S), or control miRNA (C) at a concentration of 20 nM. *P<0.05; **P<0.01; ***P<0.001; ****P<0.0001; ns, not significant; a one-way ANOVA with Dunnett's post hoc test. Data are presented as means ± SDs (n=3). (**C**) Immunoblot analysis of apoptotic markers (cleaved caspase 3, Cl-Casp3; PARP) and a proliferation marker (p-Histone H3) in BC cells treated with miR-145WT (WT), miR-145S1 (S1), or control miRNA (C) at 20 nM for 72 hours.

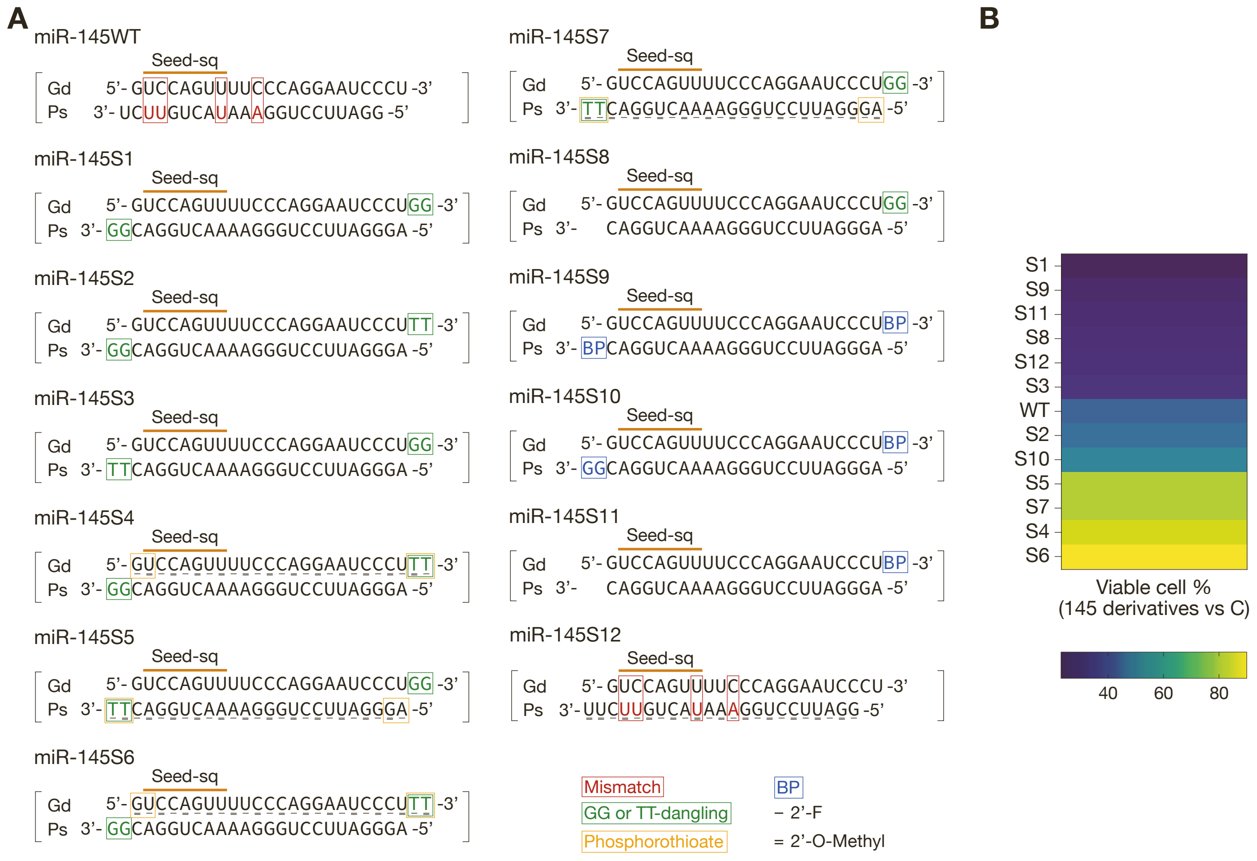

Figure S3

(**A**) Sequences and structures of miR-145 derivatives. Each miR-145 derivative contained different chemical or sequence modifications, including full-match passenger sequences, GG/TT-dangling, phosphorothioate, benzopyrene (BP), 2'-fluorine (2'-F), and 2'-O-Methyl. (**B**) Heatmap showing the relative viable cell percents of T24 cells treated with miR-145WT (WT) or miR-145 derivatives (S1-12). Viable cell percents were normalized to the viable cell counts of T24 cells treated with control miRNA.

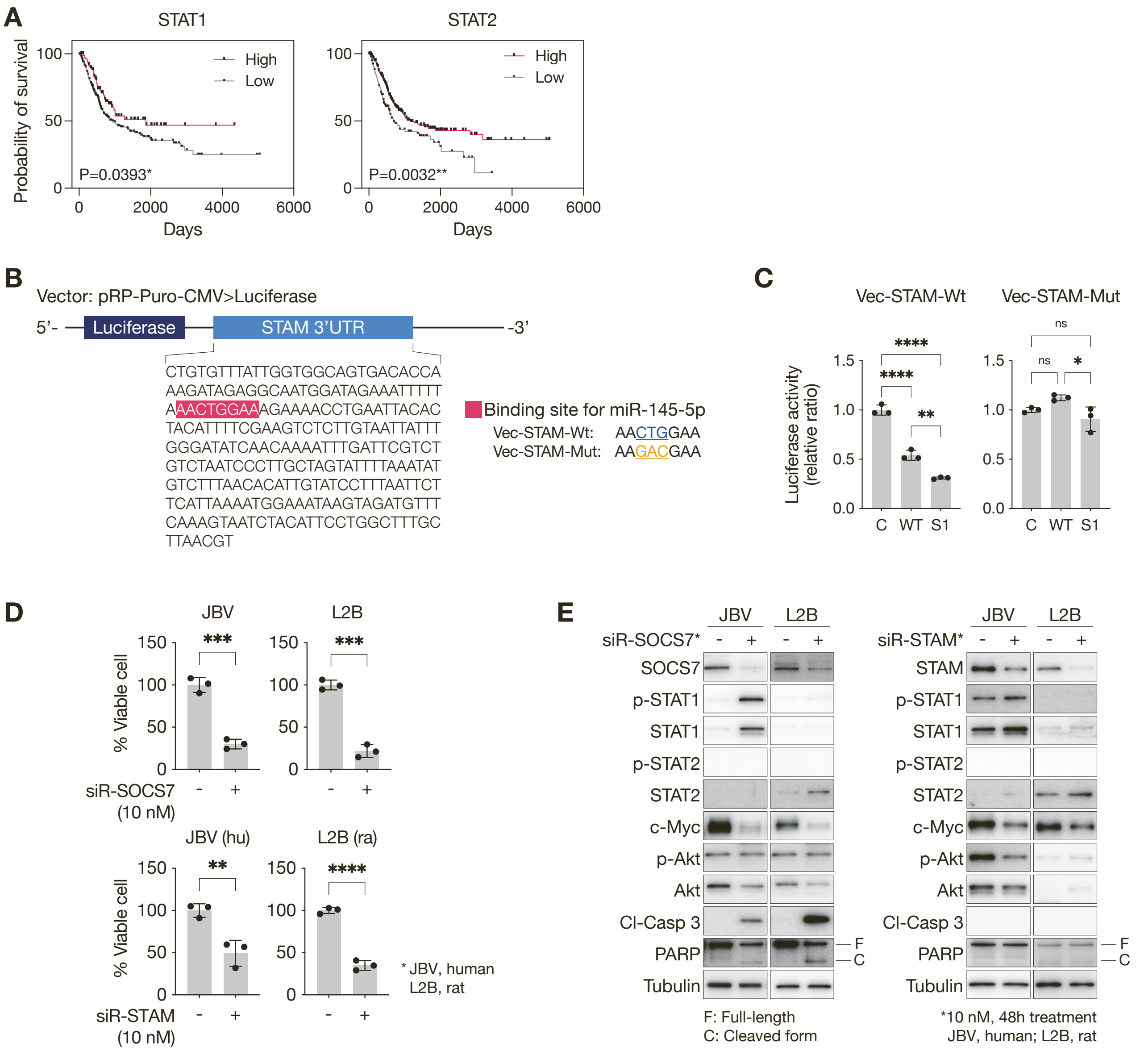

Figure S4

(**A**) Kaplan-Meier survival curves for BC patients with high and low STAT1/2 expression levels. Survival data were obtained from the TCGA database. *P<0.05; **P<0.01; the Log-rank test. (**B**) Sequences and structures of vectors constructed for the luciferase assay. The sequences highlighted in red are the binding sites for miR-145-5p in the STAM 3'UTR. Vec-STAM-Wt and Vec-STAM-Mut contained wild-type and mutant miR-145 binding sites, respectively. (**C**) Results of the luciferase assay. T24 cells were co-treated with 100 ng/mL Vec-STAM-Wt/Vec-STAM-Mut and miR-145WT (WT), miR-145S1 (S1), or control miRNA (C) at 20 nM. Data are presented as means ± SDs (n=3). *P<0.05; **P<0.01; ****P<0.0001; ns, not significant; a one-way ANOVA with Dunnett's post hoc test. (**D**) Viable cell percent among human and rat BC cells treated with miR-SOCS7, siR-STAM, or control miRNA at 10 nM for 48 hours. (**E**) Immunoblot analysis of IFN signaling pathways (p-STAT1/2, STAT1/2, SOCS7, and STAM), miR-145-associated oncogenes (c-Myc, p-Akt, and Akt), and apoptotic markers (cleaved caspase 3, Cl-Casp3; PARP) in BC cells treated for 48 hours with 10 nM of control miRNA and siR-SOCS7 or siR-STAM. Human (JBV) and rat (L2B) cells expressing endogenous c-Myc were used in the analysis to evaluate the association between SOCS7/STAM-mediated IFN signaling and c-Myc expression. Tubulin was used as the loading control.

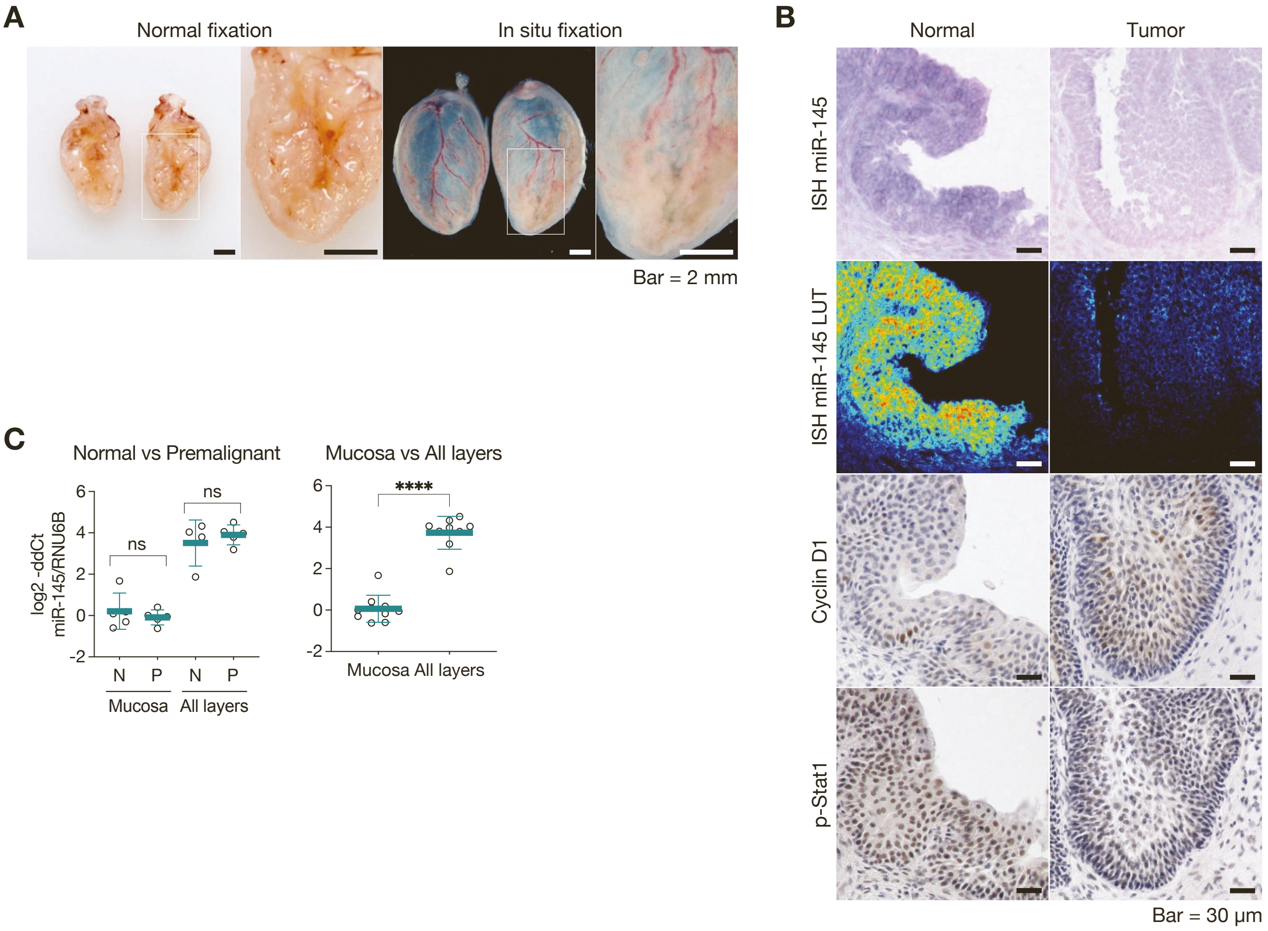

Figure S5

(**A**) Representative gross images of bladders fixed with the in situ fixation protocol or normal fixation protocol. Scale bars: 2 mm. (**B**) Representative images of miR-145 ISH and IHC for premalignant markers (Cyclin D1 and p-STAT1) in BiPLs and normal bladder tissues. A color lookup-table (LUT) was used to illustrate NBT-BCIP signal intensities; high and low expression levels are colored in red and blue to black, respectively. Scale bars: 30 μm. The simplified version is available in Figure 2F. (**C**) miR-145 expression levels in whole tissue lysates measured by miRNA RT-qPCR. Samples included rat BBN-induced premalignant lesions (P) and morphologically normal urothelial tissues (N) with (All layers) or without smooth muscle layers (Mucosa). ****P<0.0001; ns, not significant; a 2-tailed unpaired Student's t-test. Data are presented as means ± SDs. Mucosa-N, n=5; Mucosa-P, n=5; All layer-N, n=4; All layer-P, n=5.

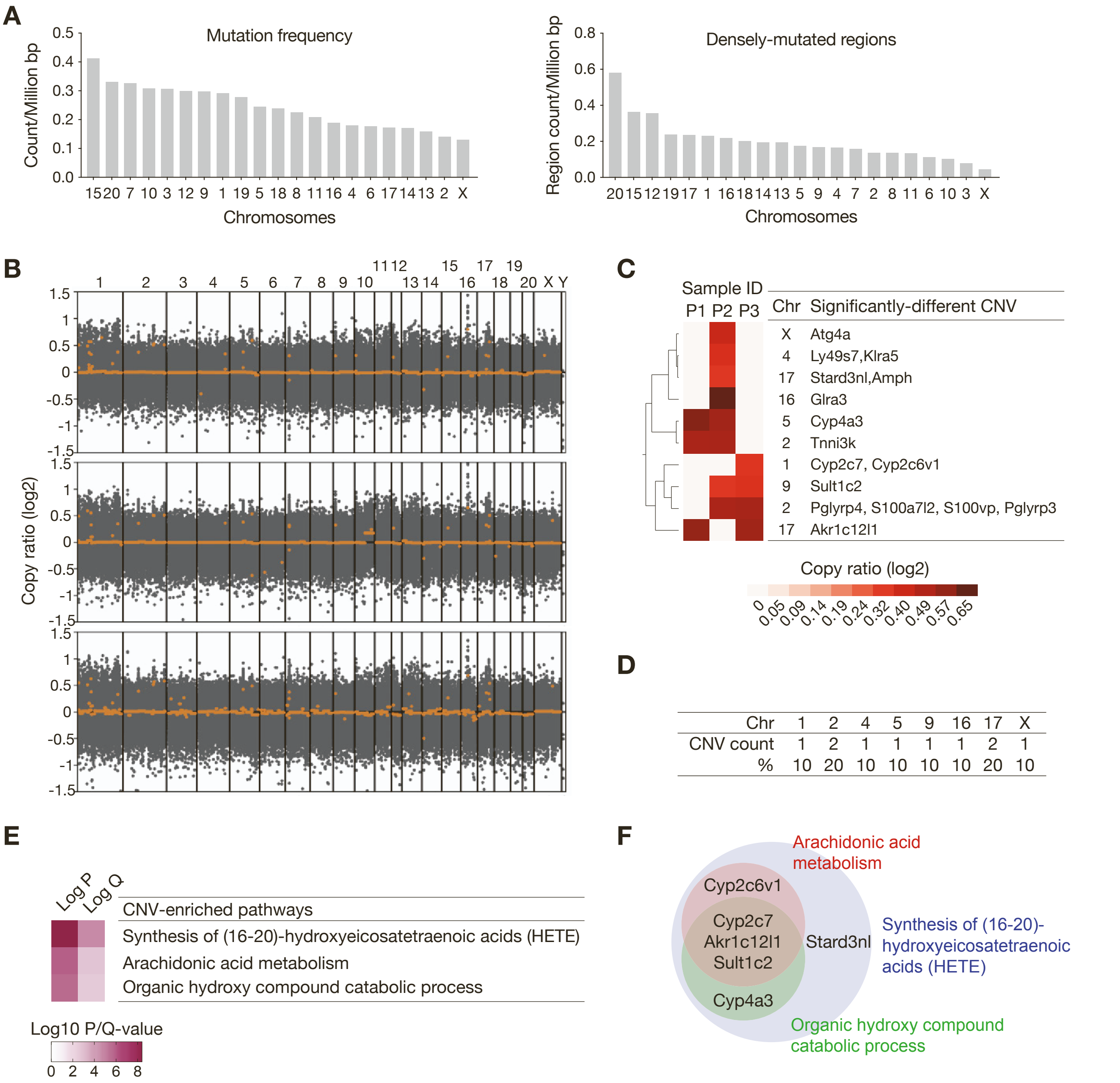

Figure S6

(**A**) Mutation frequencies and counts of densely-mutated regions on each chromosome. The counts of mutations and densely mutated regions were normalized to the length of each chromosome (per million base pairs). (**B**) Copy ratios of genes on each chromosome in rat BBN-induced premalignant lesions (BiPLs). (**C**) Copy number variants (CNVs) significantly altered in BiPL. Significantly altered CNVs were defined as those with FC > 1.25 and P-values less than 0.001. (**D**) CNV counts and percentages on each chromosome. (**E**) Enriched pathways associated with CNVs. An enrichment analysis was performed with Metascape. Significantly altered pathways were defined as the KEGG/Reactome/GO biological process pathways with P-values (Log P) ≤ 0.01 and Q-values (Log Q) ≤ 0.01. (**F**) A Venn diagram showing the names and overlap of CNVs associated with the enriched pathways.

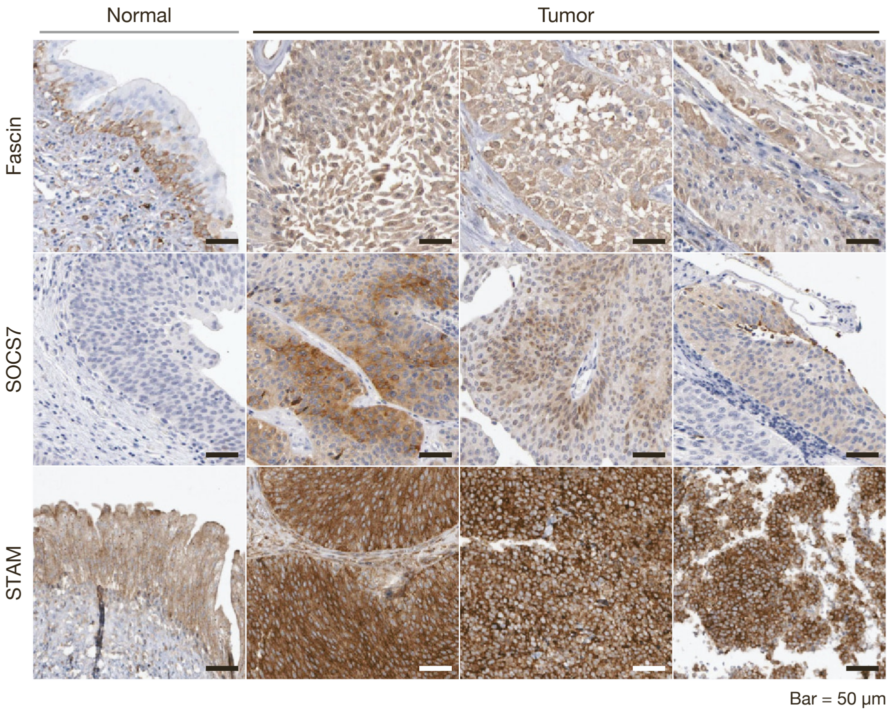

Figure S7

Representative images of BC (tumor) and normal tissues (normal) in the tissue microarray immunohistochemically stained for Fascin, SOCS7, and STAM. Images were obtained from the Human Protein Atlas. Scale bars: 50 μm.

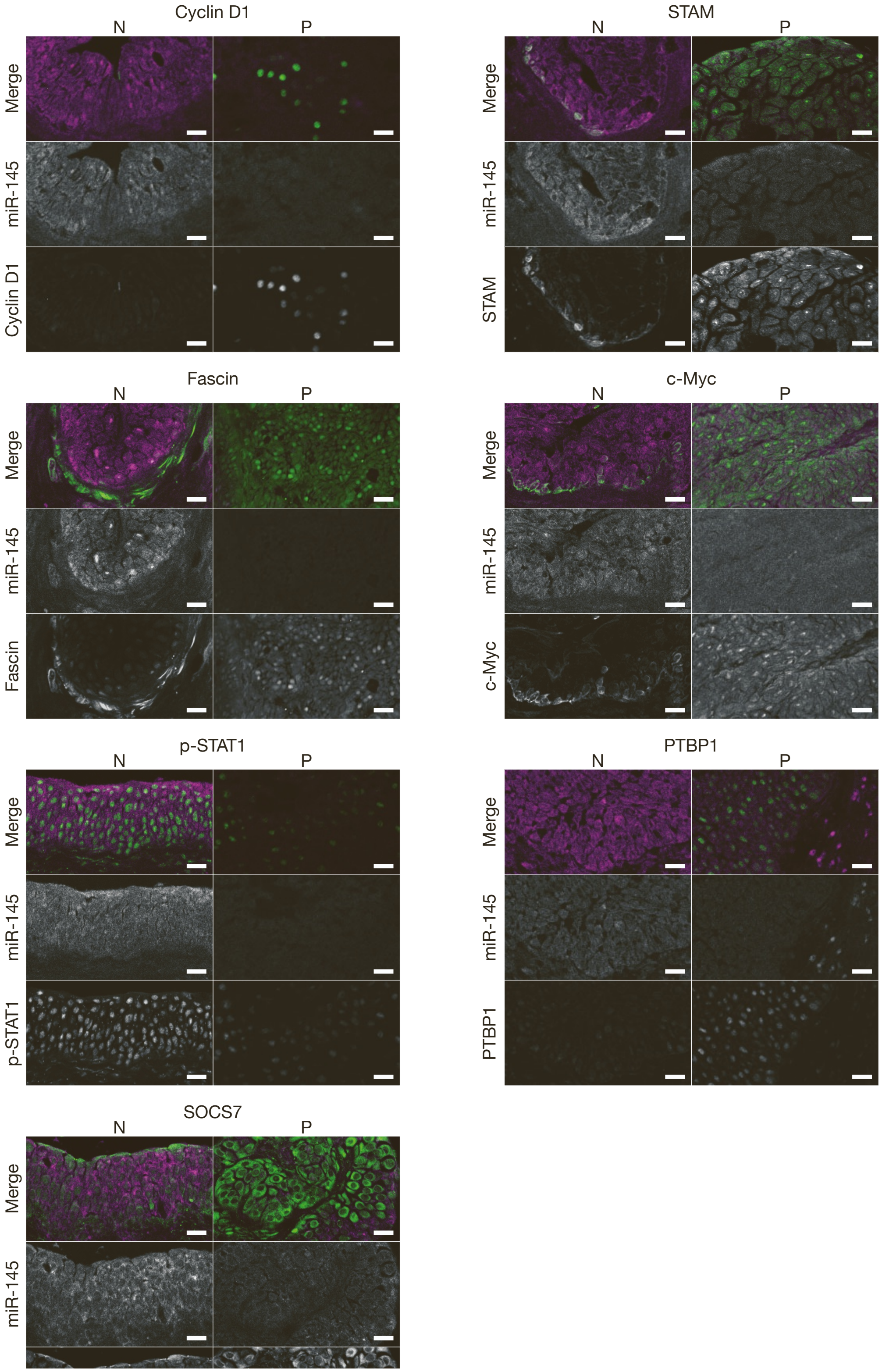

Figure S8

Representative images of each fluorescence signal from miR-145 in situ hybridization-immunohistochemistry double staining (Figure 6B). N, normal tissue; P, premalignant tissue. Scale bars: 20 μm. Data are representative of n=8.

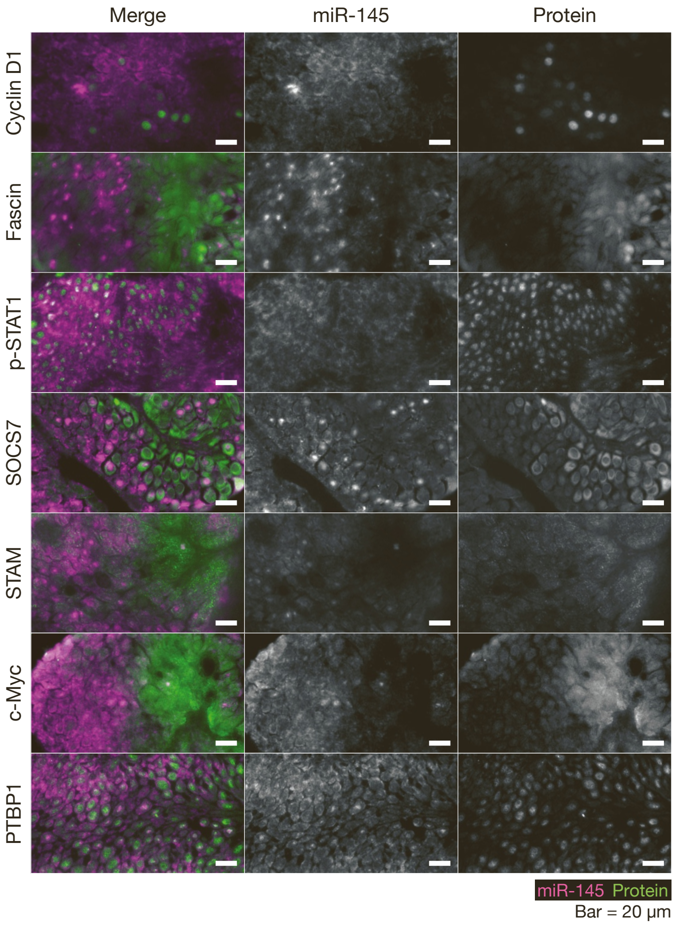

Figure S9

Representative images of each fluorescence signal from miR-145 in situ hybridization-immunohistochemistry double staining showing the intralesional gradation of miR-145 and BC-associated marker expression (Figure 6C). Data are representative of n=8. Scale bars: 20 μm.

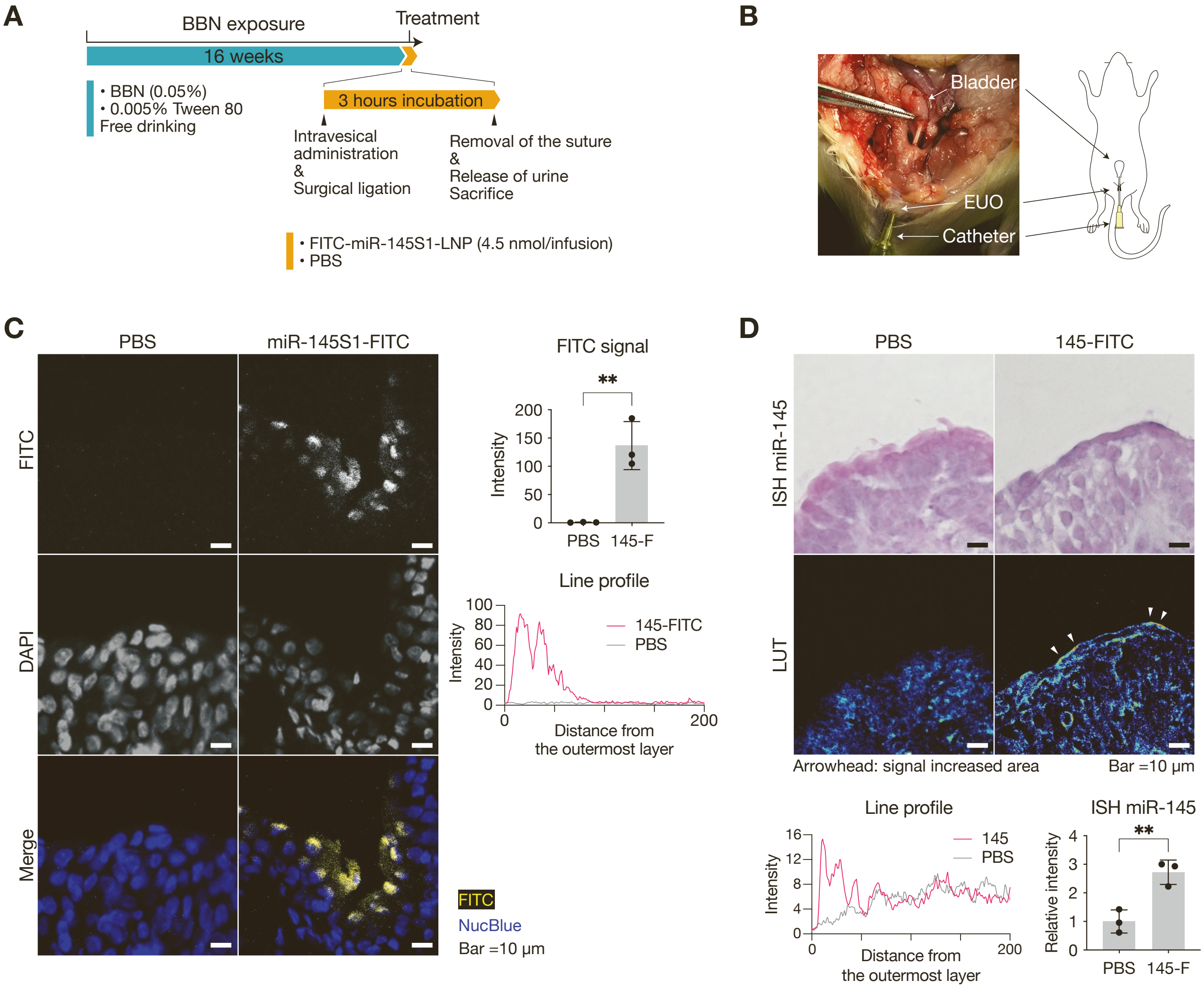

Figure S10

(**A**) Protocol for experiments to test the delivery potential of miR-145S1-LNPs in the rat BBN-induced premalignant lesion (BiPL) model. (**B**) Schematic illustration of the method of intravesical treatment. Agents were delivered into the bladder with a 24 G catheter. The agents were retained in situ for 3 hours by surgical suturing of the external urethral orifice (EUO). (**C**) Representative confocal images showing FITC signals in urothelial cells treated with FITC-labeled miR-145S1-LNPs or PBS. Signal intensities and line profiles are shown along with confocal images. Note the strong positive signals in urothelial cells in the BiPL sample. Scale bars: 10 μm. ***P<0.001; ns, not significant; a 2-tailed unpaired Student's t-test. Data are presented as means ± SDs (n=3). (**D**) Representative images of in situ hybridization (ISH) staining for miR-145 in urothelial cells treated with FITC-labeled miR-145S1-LNPs or PBS. Lookup-table (LUT) images indicating signal intensities are shown along with ISH images. Areas with an increased miR-145 signal are indicated with arrowheads.

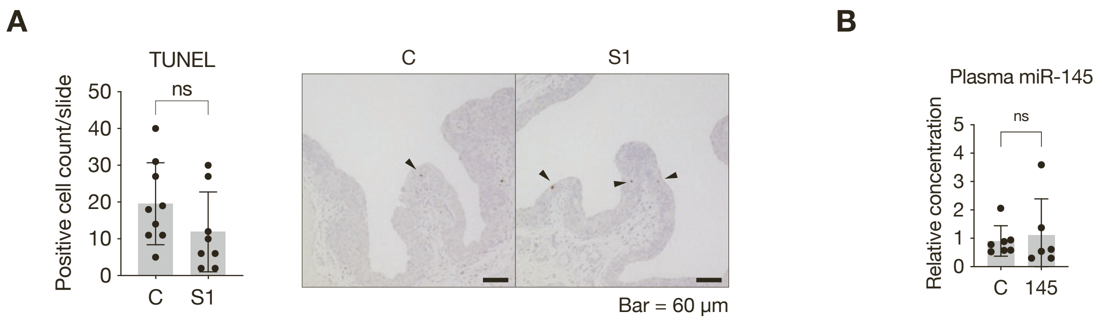

Figure S11

(**A**) Representative images and counts of TUNEL-positive cells in rat BBN-induced premalignant lesions (BiPLs) treated with miR-145S1-LNPs (S1, n=8) or ctl-RNA-LNPs (C, n=9). Scale bars: 60 μm. ns, Not significant; a 2-tailed unpaired Student's t-test. Data are presented as means ± SDs. (**B**) Plasma miR-145 levels in rats treated with control miRNA-LNP or miR-145S1-LNP in the protocol shown in Figure 7B. ns, Not significant; a 2-tailed unpaired Student's t-test. Data are presented as the mean ± SD.

Table S1 List of primers used

Primer name	Target gene	Species	Foward/Reverse	Sequence (5'-3')
hu_IFNB1_F1	IFNB1	Human	Foward	AGTAGGCGACACTGTTCGTG
hu_IFNB1_R1	IFNB1	Human	Reverse	GCCTCCCATTCAATTGCCAC
hu_XAF1_F1	XAF1	Human	Foward	GACTTCTCGGTGTGCAGGAA
hu_XAF1_R1	XAF1	Human	Reverse	CCATGGTTTCCTTGGGGACA
hu_PML_F1	PML	Human	Foward	GCCCCGTCATAGGAAGTGAG
hu_PML_R1	PML	Human	Reverse	AACGCGTTCCTCTGCCTC
hu_AIM2_F1	AIM2	Human	Foward	AGTCACCTAGGTTATTTGGGCA
hu_AIM2_R1	AIM2	Human	Reverse	CGGGTCTGCCACCTTCTTTT
hu_IRF7_F1	IRF7	Human	Foward	AGCTGTGCTGGCGAGAAG
hu_IRF7_R1	IRF7	Human	Reverse	TGGAGTCCAGCATGTGTGTG
hu_OAS1_F1	OAS1	Human	Foward	GCAGAAAGAGGGCGAGTTCT
hu_OAS1_R1	OAS1	Human	Reverse	TGGTACCAGTGCTTGACTAGG
hu_OAS2_F1	OAS2	Human	Foward	TCCGATGGTACCCTTGTCCT
hu_OAS2_R1	OAS2	Human	Reverse	GCCAGCACCTCGAAAGAGAT
hu_OAS3_F1	OAS3	Human	Foward	GTACCACCAGGTGTGCCTAC
hu_OAS3_R1	OAS3	Human	Reverse	AGGGTCCTCGAAGCCATAGT
hu_ACTB_F1	ACTB	Human	Foward	GCCTCGCCTTTGCCGA
hu_ACTB_R1	ACTB	Human	Reverse	GAATCCTTCTGACCCATGCC
hu_GAPDH_F1	GAPDH	Human	Foward	AATGGGCAGCCGTTAGGAAA
hu_GAPDH_R1	GAPDH	Human	Reverse	GCGCCCAATACGACCAAATC
hu_TBP_F1	TBP	Human	Foward	TGACCCAGGGTGCCATGA
hu_TBP_R1	TBP	Human	Reverse	GGGTCAGTCCAGTGCCATAA
ra_Ifnb1_F1	Ifnb1	Rat	Foward	CTATGGAGGTGATGCACCCG
ra_Ifnb1_R1	Ifnb1	Rat	Reverse	TCGTGGATGTCACCCAAGTC
ra_XAF1_F1	Xaf1	Rat	Foward	CTGCAACCAGCGCGTTATAC
ra_XAF1_R1	Xaf1	Rat	Reverse	TTAGGCTTGCCCTTTGCACT
ra_PML_F1	Pml	Rat	Foward	TGTGGCAAGTGCTTTGATGC
ra_PML_R1	Pml	Rat	Reverse	GGTGGTATTGTGGAGTGGGG
ra_AIM2_F1	Aim2	Rat	Foward	GTTTCAAAGTGAAAGGAGGCAGT
ra_AIM2_R1	Aim2	Rat	Reverse	GTCCTCAATTCGGGGACTGG
ra_IRF7_F1	Irf7	Rat	Foward	TTCAGCCGTAGGGATCTGGA
ra_IRF7_R1	Irf7	Rat	Reverse	AAGATAAAGCGCCCTGTGCT
ra_OAS1_F1	Oas1	Rat	Foward	GAAGTGCCGGTGGATGAGG
ra_OAS1_R1	Oas1	Rat	Reverse	CCCTCACTGTGTCAAGGTCC
ra_OAS2_F1	Oas2	Rat	Foward	AGGCAAACCTCAAACCCGAT
ra_OAS2_R1	Oas2	Rat	Reverse	TTTCCGACCATAGGAGCCAC
ra_OAS3_F1	Oas3	Rat	Foward	AGCTTCACGCTGACATCCAA
ra_OAS3_R1	Oas3	Rat	Reverse	TAGCTCTGGATGAGGTCCGT
ra_ACTB_F1	Actb	Rat	Foward	CGCGAGTACAACCTTCTTGC
ra_ACTB_R1	Actb	Rat	Reverse	CGCAGCGATATCGTCATCCA
ra_GAPDH_F1	Gapdh	Rat	Foward	GCATCTTCTTGTGCAGTGCC
ra_GAPDH_R1	Gapdh	Rat	Reverse	TACGGCCAAATCCGTTCACA
ra_TBP_F1	Tbp	Rat	Foward	GGCGGGGTCATGAAATCCAG
ra_TBP_R1	Tbp	Rat	Reverse	TCGTCACGCACCATGAAACA

Table S2 List of antibodies used

Antigen	Antibody Clone	Clonality	Vendor	Cat. No.	Host Species
Cleaved caspase 3	5A1E	Monoclonal	CST	9664	Rabbit
PARP	-	Polyclonal	CST	9542	Rabbit
p-Histone H3 (S10)	D2C8	Monoclonal	CST	3377	Rabbit
p-STAT1 (Ser727)	D3B7	Monoclonal	CST	8826	Rabbit
STAT1	-	Polyclonal	CST	9172	Rabbit
p-STAT2 (Tyr690)	D3P2P	Monoclonal	CST	88410	Rabbit
STAT2	Y141	Monoclonal	abcam	ab32367	Rabbit
SOCS7	-	Polyclonal	Invitrogen	PA5-98167	Rabbit
STAM	-	Polyclonal	CST	13053	Rabbit
c-Myc	Y69	Monoclonal	abcam	ab32072	Rabbit
Cyclin D1	E3P5S	Monoclonal	CST	55506	Rabbit
Cyclin A2	EPR17351	Monoclonal	abcam	ab181591	Rabbit
FGFR3	E-7	Monoclonal	Santa Cruz	sc-390423	Mouse
PTBP1	E5O2S	Monoclonal	CST	72669	Rabbit
Fascin	EP5902	Monoclonal	abcam	ab126772	Rabbit
p53 (rodent-specific)	D2H9O	Monoclonal	CST	32532	Rabbit
HRAS	OTI1D9	Monoclonal	LSBio	LS-C174464	Mouse
KRAS	2C1	Monoclonal	LSBio	LS-C175665	Mouse
p-Akt (S473)	D9E	Monoclonal	CST	4060	Rabbit
Akt	-	Polyclonal	CST	9272	Rabbit
PIK3CA	C73F8	Monoclonal	CST	4249	Rabbit
p-ERK1/2 (T202/Y204)	D13.14.4E	Monoclonal	CST	4370	Rabbit
ERK1/2	137F5	Monoclonal	CST	4695	Rabbit
α-Tubulin	-	Polyclonal	MBL	PM054	Rabbit
